# CSB-independent, XPC-dependent transcription-coupled repair in *Drosophila*

**DOI:** 10.1073/pnas.2123163119

**Published:** 2022-02-25

**Authors:** Nazli Deger, Xuemei Cao, Christopher P. Selby, Saygin Gulec, Hiroaki Kawara, Evan B. Dewey, Li Wang, Yanyan Yang, Sierra Archibald, Berkay Selcuk, Ogun Adebali, Jeff Sekelsky, Aziz Sancar, Zhenxing Liu

**Affiliations:** ^a^Department of Biochemistry and Biophysics, University of North Carolina at Chapel Hill, Chapel Hill, NC 27599;; ^b^Department of Biology, University of North Carolina at Chapel Hill, Chapel Hill, NC 27599;; ^c^Molecular Biology, Genetics, and Bioengineering Program, Faculty of Engineering and Natural Sciences, Sabanci University, 34956 Istanbul, Turkey

**Keywords:** XPC, transcription-coupled repair, XR-seq

## Abstract

*Drosophila melanogaster* has been extensively used as a model system to study ionizing radiation and chemical-induced mutagenesis, double-strand break repair, and recombination. However, there are only limited studies on nucleotide excision repair in this important model organism. An early study reported that *Drosophila* lacks the transcription-coupled repair (TCR) form of nucleotide excision repair. This conclusion was seemingly supported by the *Drosophila* genome sequencing project, which revealed that *Drosophila* lacks a homolog to CSB, which is known to be required for TCR in mammals and yeasts. However, by using excision repair sequencing (XR-seq) genome-wide repair mapping technology, we recently found that the *Drosophila* S2 cell line performs TCR comparable to human cells. Here, we have extended this work to *Drosophila* at all its developmental stages. We find TCR takes place throughout the life cycle of the organism. Moreover, we find that in contrast to humans and other multicellular organisms previously studied, the XPC repair factor is required for both global and transcription-coupled repair in *Drosophila*.

## Significance

We have discovered that *Drosophila*, which does not have the canonical TCR homologs, does nevertheless carry out TCR as efficiently as organisms that do. Furthermore, using the XR-seq and in vivo excision assay we have also shown that both global repair and TCR in *Drosophila* are dependent on the XPC protein and in that regard, *Drosophila* excision repair is more similar to the monocellular eukaryotic yeast repair system than it is to multicellular eukaryotes. Finally, we have generated genome-wide single nucleotide repair maps of *Drosophila* for CPDs, (6-4) photoproducts, and cisplatin-d(GpG) adducts that should be a useful source for investigators working on DNA damage, repair, and mutagenesis in *Drosophila*.

Nucleotide excision repair (excision repair) is a universal DNA repair system in the biological world. It removes DNA damage by making dual incisions bracketing the lesion to generate oligonucleotides 12 to 13 nucleotides (nt) in length in prokaryotes and 24 to 32 nt in length in eukaryotes (1–3). While originally discovered as a repair system for ultraviolet (UV)-induced DNA damage, it was later shown to remove all bulky base lesions caused by carcinogens such as benzo(*a*)pyrene and chemotherapeutic drugs such as cisplatin. The mechanism of excision repair has been investigated in considerable detail in *Escherichia coli*, *Saccharomyces cerevisiae*, and humans. Genomics studies have revealed that bacteria have homologs of the excision repair genes of *E. coli*, and eukaryotes have excision repair genes homologous to yeast and human genes. However, only limited work has been done on excision repair in organisms other than *E. coli*, yeast, and humans. In particular, the lack of mechanistic repair studies on excision repair in *Drosophila* is striking because work with *Drosophila* has examined chemical- and radiation-induced mutagenesis, double-strand break repair, and recombination.

Recently, we developed a method for genome-wide high-resolution analysis of nucleotide excision repair, called XR sequencing (XR-seq) (4, 5), which makes it possible to analyze excision repair in a wide range of organisms, including *Drosophila*, in which bulk biochemical approaches are difficult. This method has made it possible to examine with unprecedented sensitivity both pathways of excision repair, namely, global genomic repair, which repairs damage throughout the genome, and transcription-coupled repair (TCR), which repairs transcription-blocking damage in the template strand of genes at an accelerated rate (6). In a preliminary study, we used XR-seq with the *Drosophila* S2 cell line to demonstrate that these cells do perform TCR, in contrast to the generally held view that *Drosophila* lacks TCR, which was based on low-resolution repair assays and was seemingly supported by the fact that *Drosophila* lacks a CSB homolog, which is essential for TCR in humans and yeast (7–12). This unexpected finding led us to investigate excision repair in *Drosophila* in more detail.

Here, we report single nucleotide resolution maps for excision repair of UV damage in *Drosophila* at different developmental phases: embryo, larva, pupa, and adult. Our data confirm and extend the finding of TCR in *Drosophila* S2 cells to the organismal level, and in response to cisplatin damage, and unexpectedly also show that in contrast to humans and *Arabidopsis*, in *Drosophila* both global repair and TCR are dependent on the XPC damage recognition protein. In this regard, *Drosophila* is more similar to budding and fission yeasts than to any other multicellular organisms tested.

## Results

### Excision Repair of Different Damage Types in the *Drosophila* S2 Cell Line.

**Fig. 1.**
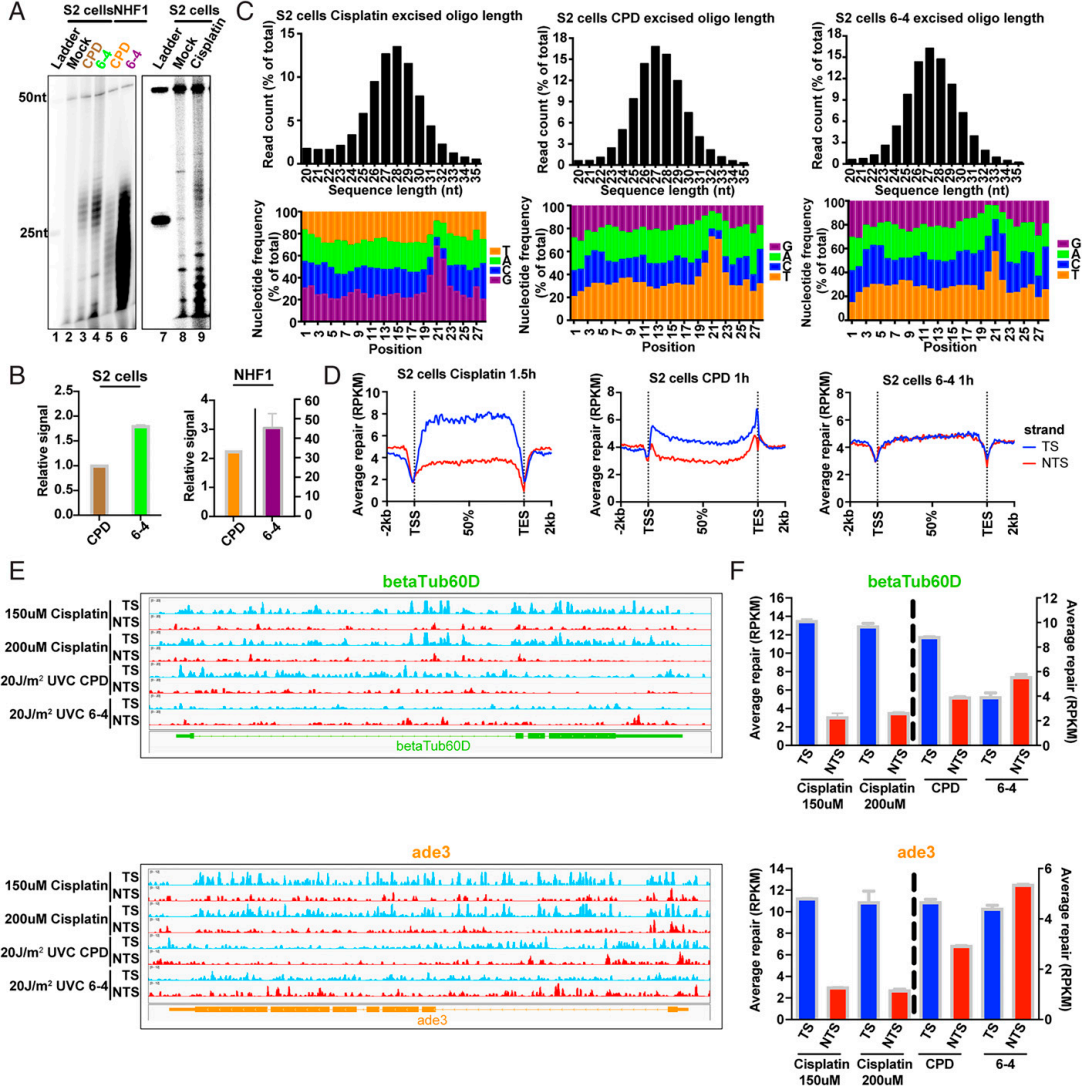
Repair of cisplatin-d(GpG) adducts and UV photoproducts in *Drosophila* S2 cells. (*A*) Excision assay of cisplatin adducts, CPDs, and (6-4)PPs in S2 cells and human NHF1 cells. All three adducts are excised in S2 cells predominantly as 26 to 29 mers, with less degradation in S2 cells than in NHF1 cells. (*B*) Quantitation of excision in *A*. Excision repair of (6-4)PPs predominates over CPDs in both *Drosophila* and human cells at the 1-h repair time point tested. Data points reflect means and SEs obtained from two experiments. (*C*) Characterization of XR-seq excision repair reads. Results are shown for cisplatin adducts (*Left*), CPDs (*Middle*), and (6-4)PPs (*Right*). *Top* shows frequency distribution profiles of excision products as percent of total reads (*y* axis) versus excision product length (*x* axis). In agreement with the excision assay results in *A*, reads of length 26 to 29 nt predominate. The peak read length is 28 nt for cisplatin and 27 nt for CPDs and (6-4)PPs. *Bottom* shows the 28-nt excision products, the relative frequency (*y* axis) of each nucleotide at each position (*x* axis). The 5′ end of the excision products is located at position 1. Enrichment of G residues on the *Left* indicates the likely site of platination at G-G dinucleotides, and enrichment of pyrimidine residues indicates the likely site of formation of CPDs (*Middle*) and (6-4)PPs (*Right*). With respect to these damage sites, sites of dual incision are located 6 nt 3′ from each adduct, and 20 nt 5′ from the cisplatin adducts and 19 nt 5′ from the CPDs and (6-4)PPs. (*D*) Repair of cisplatin and UV photoproducts in the TS (blue) and NTS (red). Excision product reads were mapped to the *Drosophila* genome and reads across all genes were scaled to a unit gene, which represents average repair in a *Drosophila* gene. Results are shown for cisplatin adducts (*Left*), CPDs (*Middle*), and (6-4)PPs (*Right*). Transcription-coupled repair is indicated by enhanced TS repair and is evident in cisplatin and CPD but not (6-4)PP repair. (*E*). Screenshots showing repair reads (*y* axis) across two representative housekeeping genes *betaTub60D* (*Top*) and *ade3* (*Bottom*) and (*F*) quantitation of repair in each strand of the two genes. TCR is evident with cisplatin and CPDs but not (6-4)PPs. Small antisense transcripts are present in *ade3* but not shown in *E*; apparently this antisense transcription is too weak to affect the overall TCR shown. Data points reflect means and SEs obtained from two experiments.

Previously, we reported that UV-induced cyclobutane pyrimidine dimers (CPDs) are repaired by TCR in S2 cells (7). We wished to test the generality of this finding by analyzing the repair of other DNA lesions known to be processed by nucleotide excision repair. We chose (6-4) photoproducts [(6-4)PPs] and cisplatin-d(GpG) adducts for this purpose as these damages have been extensively studied in excision repair research. First, we wanted to find out whether (6-4)PPs are repaired preferentially in *Drosophila* over CPDs as is the case in human excision repair (13–16). Fig. 1 *A* and *B* show that in both S2 cells and in a normal human fibroblast cell line, NHF1, (6-4)PPs are repaired more efficiently than CPDs. As we observed previously in S2 cells (7), the excision products (26 to 29 nt) are not readily degraded into smaller oligonucleotides, whereas we found that in the human cell lines, both photoproducts are degraded to smaller species rather rapidly (14, 17). Apparently, the S2 cells either lack the potent nuclease(s) involved in degrading the excision products or the degrading nucleases are expressed at lower levels. We also note that because of different amounts of genomic DNA in S2 and NHF1 cells, the absolute levels of excision between the two cell lines cannot be compared based on the data in this figure which is meant to address the issue of preferential repair of the two photoproducts in human and *Drosophila* cell lines, respectively. Finally, in Fig. 1*A*, lane 9 we show that the Pt-d(GpG) adduct is also excised mainly in the form of 26 to 29 oligomers (mers), similar to UV photoproduct excision in *Drosophila* and similar to cisplatin repair in mouse (18) and human cells (19, 20).

Next, we proceeded to characterize cisplatin adduct and UV photoproduct repair using the XR-seq procedure. In this assay (5), the excision products are isolated from cells, ligated to adaptors, purified with damage-specific antibodies [against cisplatin-, CPD-, or (6-4)PP-damaged DNA], repaired, amplified, and sequenced, and the sequenced reads are mapped to the genome (*SI Appendix*, Fig. S1). XR-seq was first done following treatment of S2 cells with cisplatin or UV light, and Fig. 1 *C*, *Top* shows, for each type of damage, the frequency of excision product reads as a function of their length. These results are consistent with the excision assay results in showing that the damages are excised mainly as 26 to 29 mers with a median of 28 nt (for cisplatin damage) or 27 nt (for UV damage). Fig. 1 *C*, *Bottom* shows the frequency of each base (*y* axis) at each position of the excised 28 mers (*x* axis) for cisplatin-d(GpG) adducts, CPDs, and (6-4)PPs. In each case, there is enrichment at positions 20 to 22 of G nucleotides (cisplatin) or pyrimidines (UV photoproducts). These sites are the presumptive location of damage in these excision products, and from these sites and the excision product length, the locations of incision sites may be determined: the 3′ incision made during repair is located 6 nt from the platinated residues and the UV photoproducts, and the major 5′ incision site is located 20 nt from the platinated residues and 19 nt from the photoproducts. These sites are consistent with prior studies of the dual incisions made by the excision nuclease in *Drosophila* and humans (7, 19, 20).

### Effect of Transcription on Cisplatin Adduct and UV Photoproduct Repair in S2 Cells.

The reads generated by XR-seq were mapped to the genome and analysis of TCR was done by scaling reads obtained from all genes in *Drosophila* to a “unit gene,” which thus represents the averaged repair in each strand of all *Drosophila* genes. This analysis of TCR revealed an interesting pattern: as shown in Fig. 1 *D*, *Left*, for cisplatin-d(GpG) adducts, the transcribed strand/nontranscribed strand (TS/NTS) repair ratio in the average gene body is ∼2, indicating strong TCR, considering that genes at all expression levels were included in the analysis and that the strong TCR in highly transcribed genes would be somewhat mitigated by weaker TCR in weakly transcribed genes. Nevertheless, Fig. 1 *D*, *Left* and *SI Appendix*, Fig. S2*A* convincingly show that cisplatin-d(GpG) is subject to TCR in S2 cells. Another feature noteworthy of cisplatin adduct repair is the “dip” of repair observed at transcription start sites (TSSs) and transcription end sites (TESs). These sites are known to be particularly A-T rich in the *Drosophila* genome and thus are expected to have lower than average frequency of the G-G sequence necessary for formation of the Pt-d(GpG) adduct (21), as our own simulation results confirm. It is possible that transcription initiation and termination complexes also contribute to the dips in repair by impeding damage formation and repair. Fig. 1 *D*, *Middle* and *SI Appendix*, Fig. S2*A* show a more modest TS/NTS ratio for CPD repair, comparable to what is seen in human NHF1 cells and is in agreement with our previous reports on S2 cells (4, 7, 19). We note that the relatively strong peak of CPD repair in both strands at the TES may arise from elevated CPD levels resulting from the nucleotide frequencies and sequences present (22, 23). Also, looping of genes in *Drosophila* may occur to the extent that it enhances repair of the TES by bringing the transcription-repair factor TFIIH from its location of high density at the promoter in proximity to the TES (24, 25). Finally, Fig. 1 *D*, *Right* and *SI Appendix*, Fig. S2*A* show that (6-4)PPs are not subject to substantial TCR at this resolution as is the case in human cells (4, 19), even though in human cells TCR is mediated by CSB, CSA, and related proteins, whereas *Drosophila* lacks both CSB and CSA homologs (26–31).

In Fig. 1 *E* and *F* we show XR-seq results in the form of browser screenshots of two representative housekeeping genes and quantitative plots. Several features of interest emerge. First, for both Pt-(GpG) and CPD there is clear TCR, whereas none is observed for (6-4)PPs, consistent with the results in Fig. 1*D*. Second, TCR of CPDs is particularly strong in the introns because in these particular genes the introns are A-T rich.

For our analyses of TCR, we exclude small and overlapping genes. Small genes are excluded due to the possibility of nonhomogeneous damage distribution in the two strands. In the case of overlapping genes, complications may arise. For example, a case in which the two genes are transcribed in opposite orientations is illustrated in *SI Appendix*, Fig. S2 *B* and *C*. XR-seq results show that in the region of overlap, likely due to relative transcription levels, TCR in the CG9821 gene predominates, resulting in more NTS than TS repair in CR43130.

### In Vivo Model for Excision Repair in *Drosophila*.

**Fig. 2.**
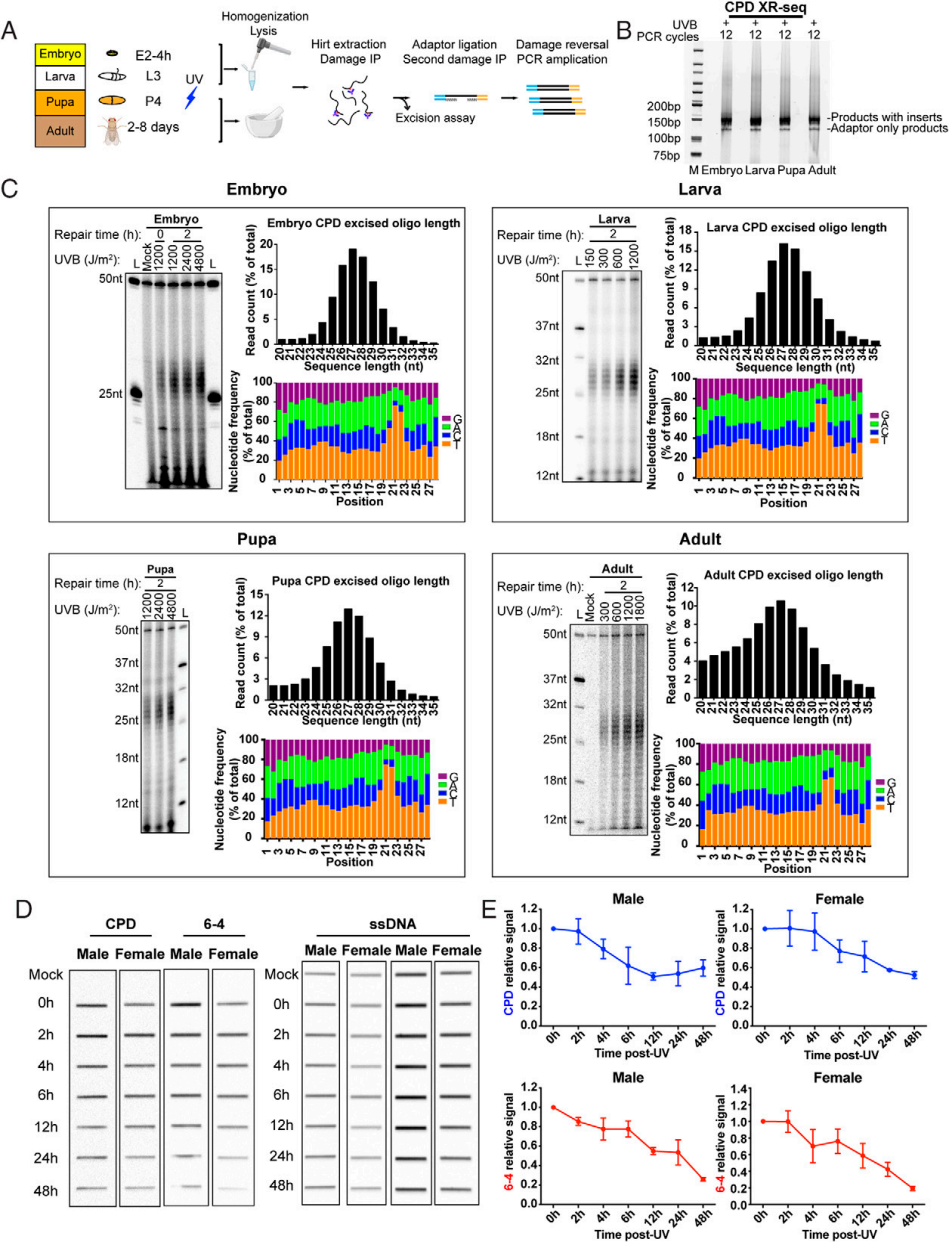
Excision repair of CPDs in *Drosophila* in vivo. (*A*) Method developed for excision and XR-seq assay of embryo, larva, pupa, and adult. *Drosophila* at each developmental stage was irradiated directly with UVB. Following repair, samples were lysed by various means to release excision products (see *Materials and Methods* and *Results*). Excision products were processed for excision assay and XR-seq as was done with cultured cells. (*B*) XR-seq libraries generated from different developmental stages. The libraries are of good quality (5). (*C*) Analysis of excision repair in vivo. For each developmental stage, excision assay results are shown alongside plots characterizing repair reads generated by XR-seq. The results show that excision product lengths peak at 26 to 28 nt among all stages in a dose-independent manner. Also, relatively little degradation of excision products is seen with *Drosophila* both in vivo and in vitro. (*D* and *E*) Slot blot analysis of CPD and (6-4)PP repair following 1,200 J/m^2^ UVB given to adults shows more rapid removal of (6-4)PPs from the genome overall. Data points reflect means and SEs obtained from two experiments.

The above results and previous studies of excision repair in *Drosophila* employed cell lines cultured in vitro (32, 33), and we were interested to know how the in vitro results extrapolated to the in vivo condition. Previous work showed that in other organisms, involvement of global and TCR are development dependent and repair may exhibit cell type–specific patterns (34–36). *Drosophila* is a complex organism with a life cycle that includes embryo (E2 [4 h]), larva (L3), pupa (P4), and adult (5 to 8 d old) developmental stages. We examined excision repair in vivo (Fig. 2*A*) by irradiating organisms at each developmental stage with ultraviolet B (UVB) and then incubating them in the dark for 2 h at 25 °C to allow excision repair to proceed in the absence of photoreactivation. Organisms at each stage were then pooled, lysed, and homogenized, and low-molecular-weight DNA was isolated and then immunoprecipitated with damage-specific antibodies following the Hirt protocol (5, 14). Samples were then processed for excision assay or processed by optimized XR-seq procedures to generate libraries for sequencing (Fig. 2*B*).

### Excision by Dual Incision and Damage Removal in *Drosophila* In Vivo.

Excision assays and frequency histogram analysis of XR-seq experiments for the four developmental stages are shown in Fig. 2*C* (CPD repair) and *SI Appendix*, Fig. S3 [(6-4)PP repair]. The results show the excision of CPDs and (6-4)PPs as 24 to 31-nt oligomers with a peak at 26 to 28 nt. Nucleotide frequency analyses show enrichment of dipyrimidines (T-T or T-C) 7 to 9 nt from the 3′ end of the in vivo 28-mer excision products. As with *Drosophila* cells cultured in vitro (Fig. 1*A*), there is relatively little degradation of the excision product in vivo. These results demonstrate good agreement between repair in the in vitro and in vivo *Drosophila* models.

To quantitatively confirm the UV adduct repair pattern over extended periods of time in female and male *Drosophila* adults, the immuno-slot blot method with damage-specific antibodies was used to measure the dynamic loss of the total amount of genomic DNA damage. Adults were irradiated with 1,200 J/m^2^ of UVB, and as seen in Fig. 2 *D* and *E*, we found that in both genders, (6-4)PP repair is almost completed within 48 h, and about half of the CPDs are removed within 48 h, consistent with repair kinetics in other organisms, including humans (14).

### Genome-Wide and TCR in *Drosophila* In Vivo.

**Fig. 3.**
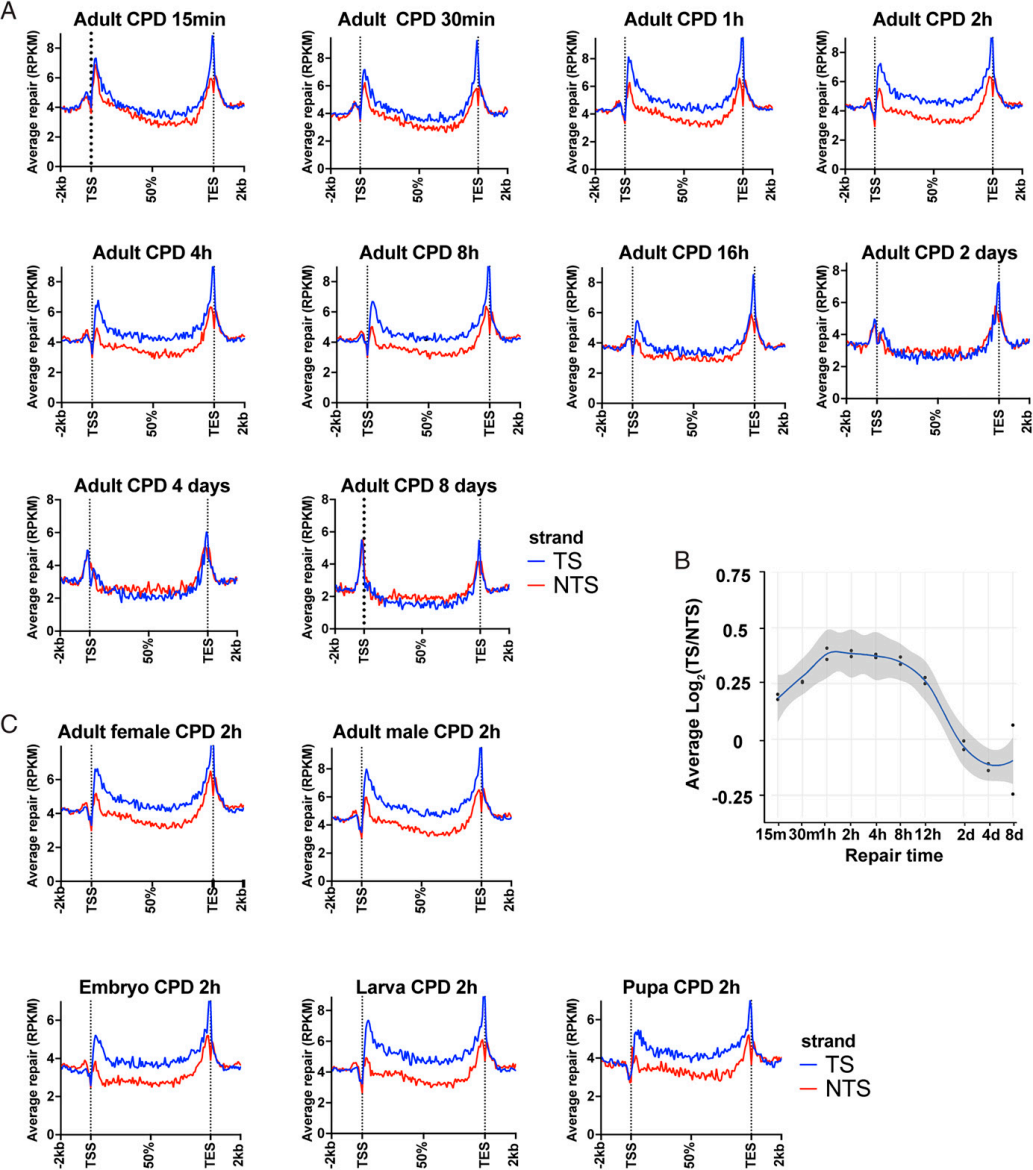
Genome-wide analysis of TCR in *Drosophila* in vivo. (*A*) *Drosophila* adult XR-seq data for the indicated time points are plotted. For each analysis, CPD repair reads were mapped to the genome, and reads from the two strands of each gene were scaled to a unit gene, which represents the average repair in each strand of genes included. Each plot includes results obtained with nonoverlapping genes longer than 1 kb and shown to be transcribed by RNA-seq (RPKM > 10). Expression levels were obtained from FlyBase RNA-seq reports (37). Reads 2 kb upstream and downstream of each gene were averaged and plotted. (*B*) Change in TCR with time. TCR is plotted on the *y* axis as average Log_2_(TS/NTS). TCR peaks at ∼2 h post-UVB and remains high until 8 to 12 h post-UV. (*C*) XR-seq results from *Drosophila* embryo, larva, pupa, and adults of each sex are plotted. TCR is evident in all samples, which were tested following 2-h repair.

We began our analysis of genome-wide repair in vivo using adult flies, and we filtered the data to analyze genes identified by FlyBase RNA-sequencing (RNA-seq) data (37) as expressed reads per kilobase per million total reads (RPKM) > 10. Time courses showing TCR in these expressed genes, ranging from 15 min to 8 d, are shown in Fig. 3 *A* and *B*. Maximal TCR appears across a broad peak centered at ∼2 h (Fig. 3*B*). As reported previously, peak repair was also seen at an early time point (30 min) in S2 cells (7), (10-min, 30-min, and 8-h repair times were tested). Interestingly, Fig. 3 *A* and *B* show that following 2-d repair in vivo, NTS repair predominates as CPDs are depleted from the template strand by rapid TCR. At this 2-d time point, ∼40% of the CPDs have been removed from the genome (Fig. 2*E*). In S2 cells, NTS repair was also clearly predominant at 16 h post-UV, and the switch from TS to NTS repair appears to have started by 8 h post-UV (7).

We next examined TCR repair patterns in different developmental stages and genders (Fig. 3*C*). This analysis revealed TCR in vivo in each stage and in both genders, as seen in S2 cells. The developmental phase dataset was then reanalyzed without filtering out weakly transcribed and nontranscribed genes, to allow comparison with the unfiltered S2 data in Fig. 1 *D*, *Middle*. The results in *SI Appendix*, Fig. S4 show weaker TCR in vivo compared to S2 cells, likely due to differences in relative transcription levels. Interestingly, more dominant repair near the TES appeared across all the repair time points compared to the human repair profiles, probably due to uniform RNAP binding of highly expressed genes (38) or the cotranscriptional cleavage termination model in *Drosophila* (22).

Finally, in plots of (6-4)PP XR-seq data for *Drosophila*, four developmental phases and both genders show that within the resolution of our assay, (6-4)PPs are not subject to substantial TCR in vivo (*SI Appendix*, Fig. S5) as is the case in S2 cells.

### Requirement for XPC in *Drosophila* Nucleotide Excision Repair.

In all multicellular eukaryotes tested, including humans and *Caenorhabditis elegans*, six repair factors XPA, RPA, XPC, TFIIH, XPG, and XPF-ERCC1 are required for global repair (3, 39). Interestingly, only five of the factors are required for TCR; XPC is dispensable (40). In fact, XPC mutants have been utilized to study exclusively TCR, in the absence of “background” global repair (4). To apply this approach to examine TCR in *Drosophila*, we used an available XPC mutant fly (G1) generated by chemical mutagenesis (31). Due to the possibility of off-target mutations, we also generated an XPC knockout (KO) by CRISPR-Cas9 genome engineering.

**Fig. 4.**
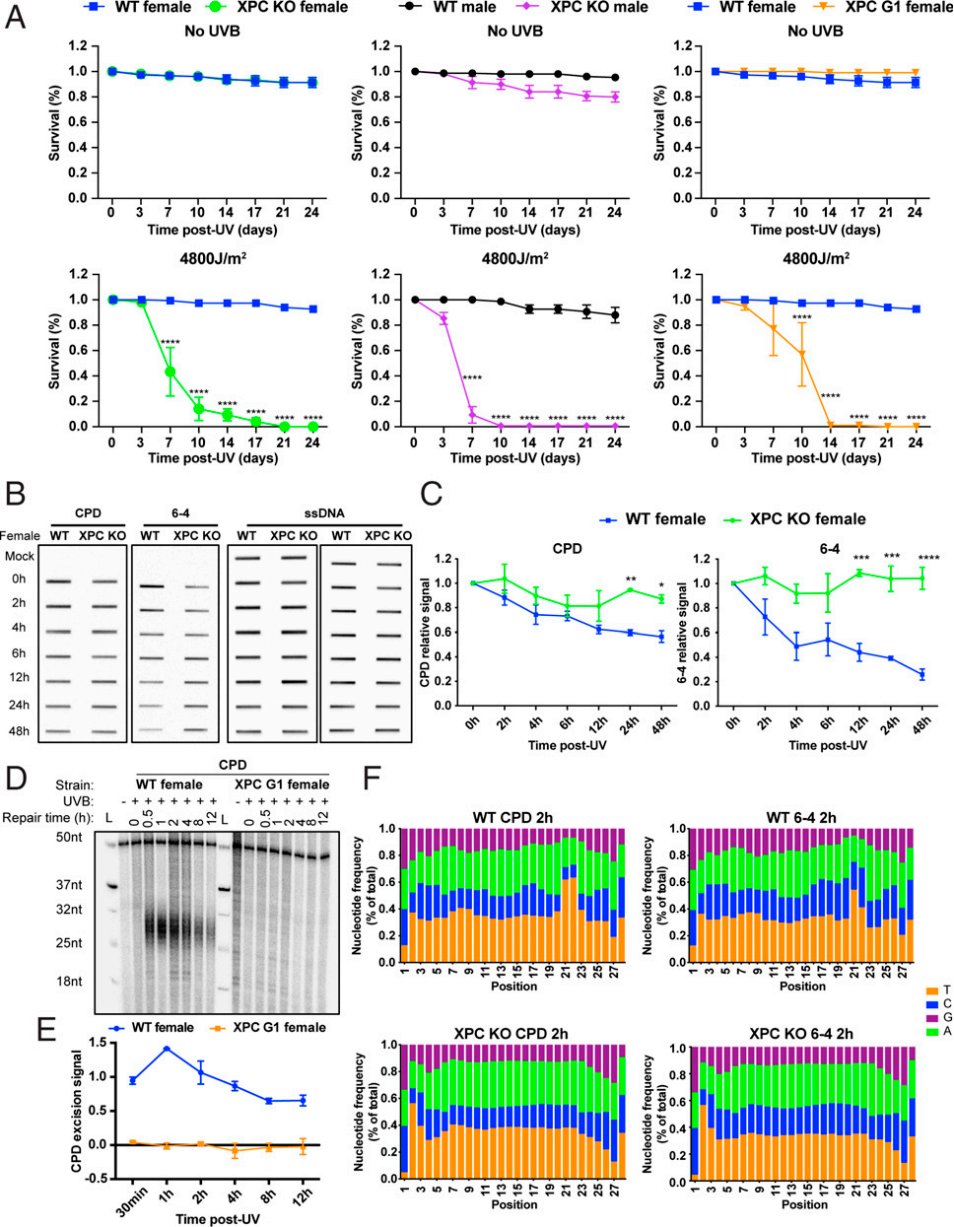
*XPC* ortholog *mus210* is required for repair in *Drosophila*. (*A*) Survival of wild type (WT, *W^1118^*), *mus210* mutant (XPC^G1^), and *mus210* knockout (XPC^KO^) adult flies without and with 4,800 J/m^2^ UVB exposure. Group data were analyzed by two-way ANOVA (Tukey’s multiple comparison test for more than two groups by using GraphPad Prism 8 software) and expressed as means ± SEM, *n* = 3. *P < 0.05, **P < 0.01, ***P < 0.001, and ****P < 0.0001 were considered to be statistically significant. XPC^G1^ males exhibited poor survival in the absence of UV and were not characterized due to this complication. (*B*) Representative slot blot showing CPD and (6-4)PP formation and repair in wild-type and XPC^KO^ female flies. The *Left* shows anti-CPD and anti-(6-4)PP immunoreactivity, and anti-ssDNA reactivity is shown to the *Right*. (*C*) Plot of slot blot results. The CPD signal was normalized to the amount of ssDNA detected in each slot. Group data were analyzed by two-way ANOVA (Šidák’s multiple comparisons test for two groups by using GraphPad Prism 8 software) and expressed as means ± SEM, *n* = 3. *P < 0.05, **P < 0.01, ***P < 0.001, and ****P < 0.0001 were considered to be statistically significant. Plotted are means and SEs from two or more experiments. (*D*) Excision assay of wild-type (WT, *W^1118^*) and XPC^G1^ (*mus210)* mutant flies. Assay was done with anti-CPD immunoprecipitation. (*E*) Plot of quantitative values from experiments represented in *D*. For plotting, results were normalized to the 50-mer labeling control oligo (see *D*), and averages and SDs from two experiments are shown. (*F*) Characterization of XR-seq excision repair reads of wild-type and XPC^KO^ adults. Wild-type excision products 28 nt in length plotted as in Fig. 2*C* show enrichment of pyrimidine residues with the same pattern as in Fig. 2*C*, indicating the likely site of formation of CPDs and (6-4)PPs. XPC^KO^ 28-nt ligation products do not show dipyrimidine enrichment, confirming our excision assay results in showing an absence of both TCR and global repair in the absence of XPC.

With the XPC mutant and knockout flies, we first conducted UV survival assays. Survival was monitored by counting the living flies every 3 or 4 d following different doses of UVB irradiation. The results with no UV and 4,800 J/m^2^ UVB (Fig. 4*A*) and several other doses (*SI Appendix*, Fig. S6) show that the XPC mutant and XPC knockout flies are quite sensitive compared to wild type. Next, we used the immuno-slot blot assay to measure loss of CPDs and (6-4)PPs following irradiation. Loss of (6-4)PP repair observed in the knockout flies was expected, since in other organisms, (6-4)PPs are repaired primarily by the XPC-dependent global repair pathway. Interestingly, CPD repair was also undetectable in XPC knockout flies, while clearly evident in wild type (Fig. 4 *B* and *C*). We then conducted excision assays using adults and embryos of the mutant and knockout strains. Surprisingly, and consistent with the slot blot results, we failed to detect any CPD or (6-4)PP excision product (Fig. 4 *D* and *E* and *SI Appendix*, Fig. S7). Finally, we conducted XR-seq using XPC adult knockout flies. Fig. 4*F* shows the frequency distribution profiles for each nucleotide at each position of the sequenced 28 mers. Clearly, the enrichment for pyrimidines at positions 21 to 22 seen in wild-type flies is absent in the knockouts, indicating that the 28 mers in the mutant are background fragments generated by shearing during DNA processing. Thus, in *Drosophila*, both global and TCR are dependent on XPC as is the case in *S. cerevisiae* and *Schizosaccharomyces pombe* (41–44).

### Tests for a Novel Protein with CSB Function in *Drosophila*.

The presence of TCR in *Drosophila*, which lacks the CS homologs, prompted an investigation into a possible alternative coupling factor. By computational analysis we identified *Arip4* and the *Domino* helicase, based upon homologies with human CSB, and by mass spectrometry we identified *Domino*, *RecQ5*, *Mit2*, *lds*, and *Brm* proteins as interacting with stalled RNAPII. Mutations in each candidate were made; however, individual mutations had no effect on TCR (*SI Appendix*, Fig. S8). Thus it appears that TCR in *Drosophila* applies a different molecular mechanism or that there might be a translocase not yet identified that might promote TCR in *Drosophila*.

## Discussion

We initiated investigations of excision repair in *Drosophila melanogaster* to address the question of TCR in this organism. We previously reported that *Drosophila* S2 cells do perform TCR even though they lack the canonical TCR factors CSA and CSB (7). The present study confirms and extends our earlier finding to include the intact *Drosophila* organism in various stages of development, and both genders. Cisplatin-d(GpG) adducts are also shown in this work to be substrates for TCR in *Drosophila*, as they are in mice (18, 45).

Overall, we find that *D. melanogaster* demonstrates considerable similarity in excision repair with other multicellular eukaryotes, including humans, mice, and *C. elegans*. Similarities include the existence of both global and transcription-coupled repair pathways, and relative rates of repair of different types of damage. The helix-distorting (6-4)PP damage is readily recognized and repaired by global repair in these organisms, and (6-4)PPs undergo global repair faster than TCR (1, 3, 16). The rate of TCR is presumably limited by the rate of transcription; TCR initiates when elongating RNAPII is blocked by damage in the template strand. TCR initially is the more rapid pathway for repair of CPDs and cisplatin adducts, because these damages are poor substrates for global repair (15, 16). Similar to the other multicellular eukaryotes, *Drosophila* exhibits a positive association between the level of transcription and TCR, and the sites of 5′ and 3′ incision made by the excision nucleases are the same among the multicellular eukaryotes.

To study *Drosophila* TCR in the absence of global repair, we employed XPC mutant flies, since in all other multicellular eukaryotes studied, XPC is required for global repair but is dispensable for TCR (5, 40). Our surprising finding that unlike other multicellular eukaryotes, *Drosophila* requires XPC for TCR is of exceptional interest. The requirement for XPC makes *Drosophila* similar to the two yeast species *S. cerevisiae* and *S. pombe* (46), the only other organisms known to require XPC for TCR. However, in contrast to *Drosophila*, yeast do possess orthologs of the CSA and CSB genes (46), which makes *Drosophila* unique in the components required for TCR.

This investigation raises the question of what the role is of XPC in TCR in *Drosophila*. The components necessary for TCR in eukaryotes have been identified and studied in considerable detail (9–12); however, the mechanism of eukaryotic TCR is not well defined for the lack of an in vitro TCR system in eukaryotes. In global repair, damage recognition and assembly of preincision complexes have been shown to be cooperative, multienzyme, multistep processes in which XPA, RPA, and XPC participate in damage recognition (15, 16, 47, 48). In addition, intermediate structures in both global repair and transcription exist in which the DNA substrate is melted or unwound in the vicinity of the substrate/damage (13, 49). Thus in *Drosophila*, there are several steps in TCR in which XPC may be required; in addition to assisting RNAPII in damage recognition, XPC may be required for assembly of preincision complexes with appropriate repair protein partners, or generation and maintenance of the appropriate DNA structure in the context of a stalled RNAPII (50). Following these preincision events, incisions are made in the damaged strand by the XPG and XPF/ERCC1 nucleases. Additional investigation is needed to characterize the role of XPC in *Drosophila* TCR.

## Materials and Methods

### Antibodies.

The antibodies used in this study were as follows: anti-CPD (Cosmo Bio Co., Ltd; NM-DND-001); anti-(6-4)PP (Cosmo Bio Co., Ltd; NM-DND-002); anti-cisplatin modified DNA (Abcam, ab-103261); anti-ssDNA (Millipore Sigma, MAB3034); and anti-RNAPII-ser2P (Abcam, ab5095).

### Plasmid Construction.

CRISPR plasmids for *Arip4* (FBgn0034976), *lds* (FBgn0002542), *Dom* (FBgn0020306), *RecQ5* (FBgn0027375), *Brm* (FBgn0000212), and *Mi-2* (FBgn0262519) were generated by using pLib6.4 (Addgene plasmid #133783). Oligos were designed by using ChopChop software (https://chopchop.cbu.uib.no) and they were synthesized by Integrated DNA Technologies. Oligos were designed for each gene and each was integrated into the pLib6.4 plasmid. Phosphorylated oligos were inserted into the *Bbs*I (NEB R05395) site of pLib6.4. Positive constructs were confirmed by Sanger sequencing (Genewiz, Inc.).

CRISPR plasmids and oligo primers are listed in *SI Appendix*, Table S1.

### Cell Lines.

S2-DGRC (*Drosophila* Genomics Resource Center) wild-type cells and S2R +-MT::Cas9 cells were obtained from the DGRC. For *Arip4*^KO^, *lds*^KO^, *Dom*^KO^, *RecQ5*^KO^, *Brm*^KO^, and *Mi-2*^KO^ S2 cell lines, S2R+-MT::Cas9 cells were transfected with the CRISPR constructs (*SI Appendix*, Table S1) by using Effectene Transfection Reagent (Qiagen 301425) according to the manufacturer’s instructions. Briefly, cells were seeded in six-well plates at a concentration of 2.0 × 10^6^ cells per milliliter in 2 mL medium and allowed at least 3 h to attach. A total of 250 ng of pLib6.4 and 250 ng of pBS130 (Addgene plasmid #26290) plasmids were diluted in 150 µL of Buffer EC (Qiagen 301425). Then, 3.2 µL enhancer was added to the mixture. After a 5-min incubation, 10 µL Effectene Transfection Reagent was added to the mixture and the mixture was incubated for 15 min at room temperature. Mixtures were then added to the cells dropwise and the cells were incubated for 4 d. After 4 d, cells were given medium with 5 µg/mL puromycin (Corning, 61-385-RA) to select for transfected cells. The efficiency of transfection and the presence of gene editing was tested with Sanger sequencing of PCR products encompassing the target site. The sequencing data were analyzed by using Synthego software (https://ice.synthego.com).

All S2 cells were cultured at 27 °C in Schneider’s medium (Gibco, 21720-024) with heat-inactivated fetal bovine serum (FBS) (Sigma, F2442-500ML) at 10%, 50 units/mL penicillin, 50 µg/mL streptomycin (Pen Strep, Gibco, 15140-122) and with 200 µg/mL hygromycin B (Roche, 10843555001) antibiotic. Cells were passaged when they reached 80 to 90% confluence.

NHF1 (normal human fibroblast) and XPC patient cell lines were described previously. All mammalian cell lines were cultured in Dulbecco’s modified Eagle’s medium (DMEM) (Gibco, 11995-065) supplemented with 10% FBS, 50 units/mL penicillin, and 50 µg/mL streptomycin and maintained at 37 °C in a 5% CO_2_ environment.

### Fly Stocks.

*Drosophila* stocks were kept at 25 °C and fed with standard cornmeal medium. The *Yw* fly line was kindly provided by Mark Peifer, University of North Carolina at Chapel Hill, Chapel Hill, NC. *W^1118^* and XPC (*mus210*)^G1^ mutant fly lines were from the J.S. laboratory (31). The XPC^G1^ mutant files were from the *W^1118^* background.

### CRISPR-Cas9 Deletion of XPC via Scarless Allele Replacement.

The endogenous XPC gene was deleted and replaced with *dsRed* using CRISPR-Cas9 genome engineering and scarless allele replacement as illustrated in *SI Appendix*, Fig. S9 and described previously (51). A plasmid containing *dsRed* under the control of the eye-expressing 3xP3 promoter and flanked with DNA homologous to 5′ and 3′ XPC flanking sequences (pGEM Δ*XPC*), and another plasmid containing 5′ and 3′ XPC gRNAs (pCFD4 *XPC* gRNA), were simultaneously injected into *Drosophila* embryos expressing Cas9 in their germline stem cells under control of the nanos promoter (Genetivision). Upon eclosion of these embryos, male progeny were screened for expression of dsRed (and likely deletion of *XPC*) in their eyes using a fluorescent microscope. These males were then mated to Pin/CyO females to isolate and balance the suspected deletion. Deletions were then further screened via genomic extraction, PCR, and sequencing of parental flies used to establish the *XPC* deletion stock to confirm the deletion. All parents contained both the correct 5′ and 3′ flanking genomic sequence, indicating that *XPC* was successfully deleted and replaced with dsRed.

### Adult Fly Survival Assay Following UVB Treatment.

*W^1118^*, XPC^G1^, and XPC^KO^ mutant flies were counted and equal numbers of each were separated into individual vials. A total of 50 flies of each sex were irradiated with doses of UVB ranging from 0 to 9,600 J/m^2^. Flies were returned to their respective vials, and the number of surviving flies was recorded every 3 or 4 d. Flies were transferred to new tubes weekly. Survival was plotted to analyze sensitivity as a function of mutation status and sex. Three independent biological replicates were performed.

### Excision Assay and XR-Seq.

For in vitro excision and XR-seq assays of *Drosophila*, S2 cells were plated in 150-mm tissue culture plates and grown to about 80% confluence. For ultraviolet C (UVC) treatment, one 150-mm plate was irradiated with 20 J/m^2^ UVC after medium was removed and then fresh medium was added. For cisplatin treatment, 3.3 mM cisplatin in 0.9% NaCl was added to the medium to dilute to 200 µM. Following predetermined repair times, plates were placed on ice and cells were harvested by scraping, washed with ice-cold PBS, pelleted, and transferred to Eppendorf tubes. The cells were then resuspended in 320 μl Tris-EDTA (TE). After the addition of 40 µL 10% sodium dodecyl sulfate (SDS), the samples were incubated at room temperature for 20 min. After incubation, 100 µL 5 M NaCl was added to the samples. The mixtures were inverted to mix and they were incubated at 4 °C overnight.

In vivo excision and XR-seq assays were performed with *Drosophila* embryos, larva, pupa, and adults. For these experiments, *Drosophila* adults were isolated with fly food containing 5% sucrose and 6% agar for 2 h during which time they laid eggs. After the adults were removed, embryos were collected and irradiated with predetermined doses of UVB and then were allowed to repair in the dark for predetermined times. Larvae and pupae were transferred to 100-mm plates and irradiated with UVB and allowed to repair in the dark. After repair, samples (embryo and larva) were collected with ice-cold phosphate-buffered saline (PBS) and washed several times. A total of 320 µL TE pH 8.0 was added to each sample and samples were ground. After grinding, 40 µL 10% SDS was added to each sample and samples were incubated at 70 °C for 30 min. After incubation, 100 µL 5 M NaCl was added to each sample and they were incubated at 4 °C overnight.

*Drosophila* adults were collected on a CO_2_ panel for UVB irradiation. Flies were then returned to their vials and allowed to repair in the dark for predetermined times. After their respective repair, pupa and adults were transferred to 15-mL Falcon tubes and frozen with liquid nitrogen. While frozen, they were ground in liquid nitrogen and the powder was transferred to individual Eppendorf tubes. Then, 320 µL TE pH 8.0 and 40 µL 10% SDS were added, and samples were incubated at 70 °C for 30 min. After incubation, 100 µL 5 M NaCl was added to the samples and they were incubated at 4 °C overnight.

After overnight incubation, samples from each developmental phase were centrifuged at high speed at 4 °C for 1 h. Supernatants were processed for excision or XR-seq assay as described previously (4, 14). Briefly, supernatants were incubated with 5 µL RNase A and then 5 µL Proteinase K, purified, and then immunoprecipitated with anti-CPD, anti-(6-4)PP, or anti-cisplatin modified DNA antibodies. Immunoprecipitates were then either 3′ end (cordycepin) or 5′ end (γ-[^32^P]ATP) radiolabeled, or 3′ end biotin labeled, and excision products were separated on 10 to 11% sequencing gels, or, for XR-seq assay, immunoprecipitates were ligated to the adaptors, purified by a second immunoprecipitation, and DNA damage was reversed (using photoreactivation for UV photoproducts, and NaCN treatment for cisplatin damage). Libraries were generated by PCR and then sequenced.

### Bioinformatics Analysis.

At least 6 million unique mapped reads were obtained for each sample. Obtained reads were aligned onto the *D. melanogaster* dm6 genome assembly. Genomic distributions of the XR-seq reads were visualized using the Integrative Genomics Viewer (IGV) as described previously (7). For plotting average repair profiles as a unit gene, we chose genes that did not overlap with any other gene and that were at least 1 kb long. With these criteria, the total number of selected genes was 6,218. Regarding developmental and sex-specific expression, expressed (RPKM > 10) genes were determined according to FlyBase RNA-seq data (37). Unit genes used to show TCR were formed by dividing each gene evenly into 100 bins from the TSS to the TES and adding 2-kb (25 bins) flanking regions to upstream of the TSS and downstream of the TES. RPKM for each bin was determined and the average RPKM of bins corresponding to the same relative location was calculated using custom Python scripts and plotted with GraphPad Prism 8 software. Frequency distributions of Log_2_(TS/NTS) were plotted using all annotated *Drosophila* genes.

For details on sequence simulations, slot blot, immunoprecipitation of RNAPII-S2 and liquid chromatography-mass spectrometry/mass spectrometry (LC-MS/MS) analysis, statistical analysis, and homology-based prediction of CSB homologs, see *SI Appendix*, *Materials and Methods*.

## Supplementary Material

Supplementary File

## Data Availability

The XR-seq raw data and the sequence data have been deposited in the Gene Expression Omnibus (GEO) (https://www.ncbi.nlm.nih.gov/geo/query/acc.cgi?acc=GSE184926) (52). Code used for analysis is available on GitHub: https://github.com/saygingulec/XRSeqAnalyses. The mass spectrometry proteomics data have been deposited in the ProteomeXchanger Consortium via PRIDE partner repository with the dataset identifier PXD028924 (53).
