# Supplementary material for: CSB-independent, XPC-dependent transcription-coupled repair in Drosophila
Source: Proc Natl Acad Sci U S A. 2022 Feb 25;119(9):e2123163119. doi: 10.1073/pnas.2123163119 (PMC8892495; doi:10.1073/pnas.2123163119)
Supplement: Supplementary File [file pnas.2123163119.sapp.pdf]

PNAS
www.pnas.org

Supplementary Information for

CSB-independent, *XPC*-dependent transcription-coupled repair in *Drosophila*

Nazli Deger*, Xuemei Cao*, Christopher P. Selby, Saygin Gulec, Hiroaki Kawara, Evan B. Dewey, Li Wang, Yanyan Yang, Sierra Archibald, Berkay Selcuk, Ogun Adebali, Jeff Sekelsky, Aziz Sancar †, Zhenxing Liu †

*These authors contributed equally to this work.
†Corresponding author email: aziz_sancar@med.unc.edu; zhenxing@ad.unc.edu.

This PDF file includes:

Supplementary information about the details of materials and methods
Figures. S1 to S9
Table S1
Legends for Figures. S1 to S9 and Table S1
SI References

Supplement information

Materials and methods

Sequence simulations

XR-seq simulations were performed to generate synthetic reads that resemble the nucleotide content of the excised oligomers. We used a tool named “boquila” for the simulations, which generates pseudo next-generation sequencing reads that overall have similar nucleotide distribution with the given sequencing file. We simulated all XR-seq samples through boquila to understand and eliminate the impact of sequence content on repair. We used fastq files as inputs after the adaptor trimming step. Boquila is available on GitHub: https://github.com/CompGenomeLab/boquila

Slot blot

Adult flies were collected on a CO2 panel and were irradiated with 1200 J/m^2 UVB. After UVB irradiation, the adults were returned to their vials to allow repair in the dark for predetermined times.

After repair, flies were ground by pestle in 320 µL TE pH 8.0. Then, 40 µL SDS (10%) was added to each sample, and samples were incubated at 70°C for 30 min. After incubation, 10 µL of 5M NaCl was added to each sample and samples were incubated at 4°C overnight. Samples were then centrifuged at 14,000 rpm at 4°C for 1 h. Supernatants were incubated with 5 µL RNaseA at 37°C for 1 h, then were incubated with 5 µL proteinase K at 60°C for 1 h, then the DNA was precipitated with ethanol. DNA was resuspended with 100 µL of water, and then purified with Qiagen PCR purification kit (Qiagen Cat. No. 28106). DNA was then quantified, and 100 ng of DNA for each sample was loaded into a well of a slot blot apparatus. DNA was transferred to a membrane and the membrane was dried in a vacuum oven at 80°C for 90 min, and then blocked at room temperature for 1 h with 5% milk in

1X PBS with 0.1% Tween (PBS-T). Later, the membrane was washed with PBS-T 3 times, 5 min each. Then, the membrane was incubated with anti-CPD or anti-(6-4) PP antibodies at 4°C overnight. Membranes were then washed with PBS-T as described above, and then incubated in secondary antibody at room temperature for 1 h. After washing as described above, membranes were developed using the Bio-Rad Western ECL Kit. DNA loading was measured using anti-ssDNA antibodies (after stripping the membrane of anti-CPD or anti-(6-4) PP antibodies by incubating blots at 120 to 200 rpm for 2 h in strip buffer [2% SDS, 62.5%Tris pH 6.8, 0.8% ß-mercaptoethanol]). Two or three independent biological replicates were done for each experiment. Repair of CPD or (6-4) PP damage was calculated and data was plotted by using GraphPad Prism 8 software.

Immunoprecipitation of RNAPII-S2 and LC-MS/MS analysis

S2-DGRC (*Drosophila* Genomics Resource Center) wild type cells were seeded in 10 R-150 plates and grown to 50-80% confluence. Medium was removed and cells were irradiated with 20 J/m^2 UVC, then fresh medium was added to cells. Cells were harvested 1 h after UV-irradiation.

Cells were incubated for 20 min on ice in IP-150 buffer (30 mM Tris pH 7.5, 130 mM NaCl, 2 mM $MgCl_2$, 0.5% Triton X-100, protease inhibitor cocktail (Roche, Cat# 11873580001)), centrifuged, and the supernatant was removed (soluble fraction). The cell pellets were lysed in IP-130 buffer with 250 U/mL benzonase nuclease (Millipore, Cat # 71205). For IP, we added 2 µL RNAPII-S2 (Abcam, ab5095) to half of the lysate, and to detect non-specifically bound proteins, we added Rabbit IgG polyclonal (Abcam, ab171870) to the other half. Samples were rotated for 2-3 h at 4 °C. Then, protein A agarose beads (Invitrogen, Cat # 15918-014) were added and samples were rotated for 2-3 h at 4° C. The beads were then washed six times with IP-130 buffer. Bound proteins were eluted by boiling in SDS sample buffer. Samples were run on a 5% acrylamide gel. The gel was subsequently stained

by Coomassie dye (Brilliant Blue G, Sigma, Cat # B0770). We conducted three independent biological replicates of each experiment.

Briefly, samples of proteins immunoprecipitated with RNAPII were fractionated on 10% SDS-PAGE gel, protein bands were tryptic digested at 37°C for 16h. Peptides were extracted and desalted with house-made C18 stageTips. Desalted peptides were dissolved in 20 µl 0.1% formic acid (Thermo Fisher) for LC-MS/MS analysis with an Easy nanoLC 1200 coupled to a Q-Exactive HFX mass spectrometer. 5 µl of peptides were loaded on to a 15 cm C18 RP column (15 cm × 75 µm ID, C18, 2 µm, Acclaim Pepmap RSLC, Thermo Fisher) and eluted with a gradient of 5-30% buffer B (80% acetonitrile in 0.1% formic acid) at a constant flow rate of 300 nl/min for 17 min followed by 30% to 40% B in 3 min and 100% B for 10 min. The Q-Exactive HFX was operated in the positive-ion mode with a data-dependent automatic switch between survey Full-MS scan (m/z 350-1400) and HCD MS/MS acquisition of the top 15 most intense ions. Survey scans were acquired at a resolution of 60,000 at m/z 200. Up to the top 15 most abundant isotope patterns with charge $\geqslant 2$ from the survey scan were selected with an isolation window of 1.4 m/z and fragmented by HCD with normalized collision energies of 27. The maximum ion injection time for the survey scan and the MS/MS scans was 100 ms, and the ion target values were set to 1e5 and 1e4, respectively. Selected sequenced ions were dynamically excluded for 20 seconds. There were three biological replicates and each sample was subjected to two technical LC-MS/MS replicates.

Mass spectra processing and peptide identification was performed using the MaxQuant software version 1.6.10.43 (Max Planck Institute, Germany). All peptide matching searches were performed against the UniProt *Drosophila melanogaster* protein sequence database (UP000000803). The mass spectrometry proteomics data have been deposited to the ProteomeXchange Consortium via PRIDE partner repository with the dataset identifier PXD028924. A false discovery rate (FDR) for

both peptide-spectrum match (PSM) and protein assignment was set at 1%. Search parameters included up to two missed cleavages at Lys/Arg on the sequence, oxidation of methionine, and protein N-terminal acetylation as a dynamic modification. Carbamidomethylation of cysteine residues was considered as a static modification. Data processing and statistical analysis were performed on Perseus (Version 1.6.10.50). Label-free quantification (LFQ) was performed on biological and technical replicate runs, and a two-sample t-test statistics was used to report statistically significant fold-changes (FDR=0.05, fold change >2).

Statistical analysis

Group data were analyzed by 2-way ANOVA (Tukey's multiple comparison test for more than two groups and Šidák's multiple comparisons test for two groups) (GraphPad Prism 8 software) and expressed as means ± SEM unless otherwise indicated. $^{*}p < 0.05$, $^{**}p < 0.01$, $^{***}p < 0.001$ and $^{****}p < 0.0001$ were considered to be statistically significant.

Homology-based prediction of CSB homologs

We applied two methods to predict potential candidates for TCR factors.

Phylogenetic approach: We blasted human ERCC6 protein against both *Homo sapiens* and *Drosophila melanogaster* proteomes. Then, we concatenated the protein sequences into a single FASTA-formatted file. A multiple sequence alignment was constructed from these sequences by using MAFFT (1) tool "einsi" option. We used IQtree version 2.0.6 (2) for phylogenetic tree construction with a maximum-likelihood approach. From the phylogenetic tree, we selected the sister clade containing homologs for ATRX, ARIP4, RAD54. We chose ARIP4 ortholog Q9W1A8 as a candidate because it was understudied compared to other *Drosophila melanogaster* proteins.

PSI-Blast approach: We first identified the domains of human ERCC6 protein by using CDvist algorithm (3). Then, we identified a helicase domain between residues

840 and 952. We extracted the amino acid sequence starting from residue number 953, until the end of C-terminus. We blasted the extracted sequence by using PSI-BLAST(4) algorithm against UniProt (5) *Drosophila melanogaster* proteome. We iterated the algorithm three times and Helicase *Domino* was the highest scoring hit in terms of query coverage (51%) and E-value (8e-93). Therefore, we selected Helicase *Domino* as the second candidate.

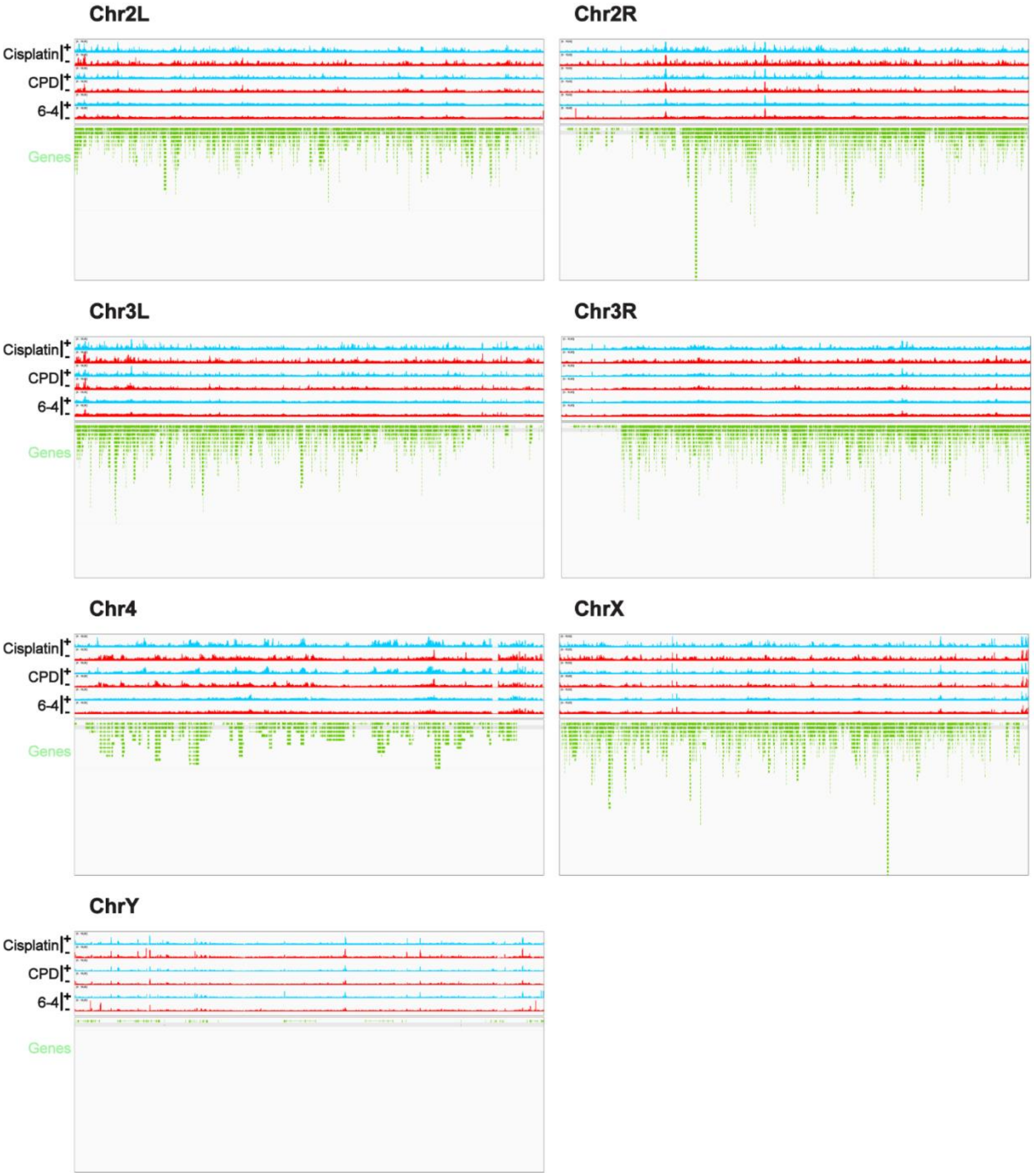

Figure S1. Repair sites mapped to the *Drosophila* genome. Distribution of the XR-seq signal, separated by strand (+, blue; -, red), for cisplatin adducts, CPDs and (6-4) PPs in S2 cells across all chromosomes of the *Drosophila* genome. Genes are shown in green.

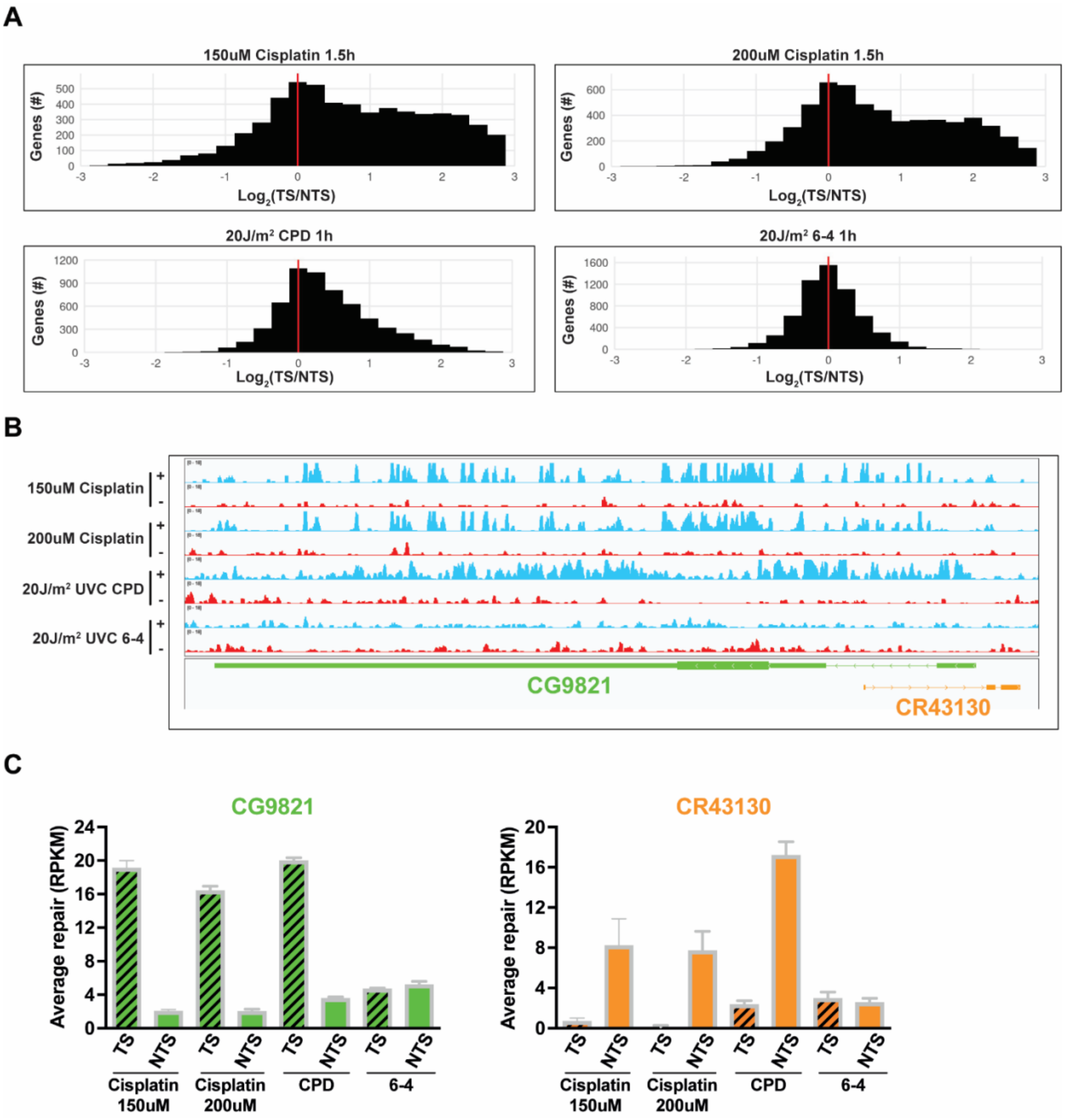

Figure S2. TCR in S2 cells following repair of cisplatin adducts and CPDs but not (6-4) PPs. **(A)** Plots of number of genes (y-axis) as a function of magnitude of TCR, expressed as $\log_2$(TS/NTS) (x-axis). The right-shifted distribution of reads seen following cisplatin adduct and CPD repair reflects the effect of rapid TCR producing more repair of TS vs NTS at the time point tested. The equal distribution

of reads in the (6-4) PP sample indicates no substantial TCR of this damage in S2 cells. **(B)** Screenshots showing repair reads (y-axis) across two antisense overlapping genes CG9821 (left) and CR43130 (right) and **(C)** Quantitation of repair in each strand of the two genes. Stronger transcription and TCR of CG9821 presumably produces the preferential NTS repair in CR43130. In **(B)**, CG9821 TS repair is in blue and NTS repair is red; for CR43130, TS is red and NTS is blue. Data points reflect means and standard errors obtained from two experiments.

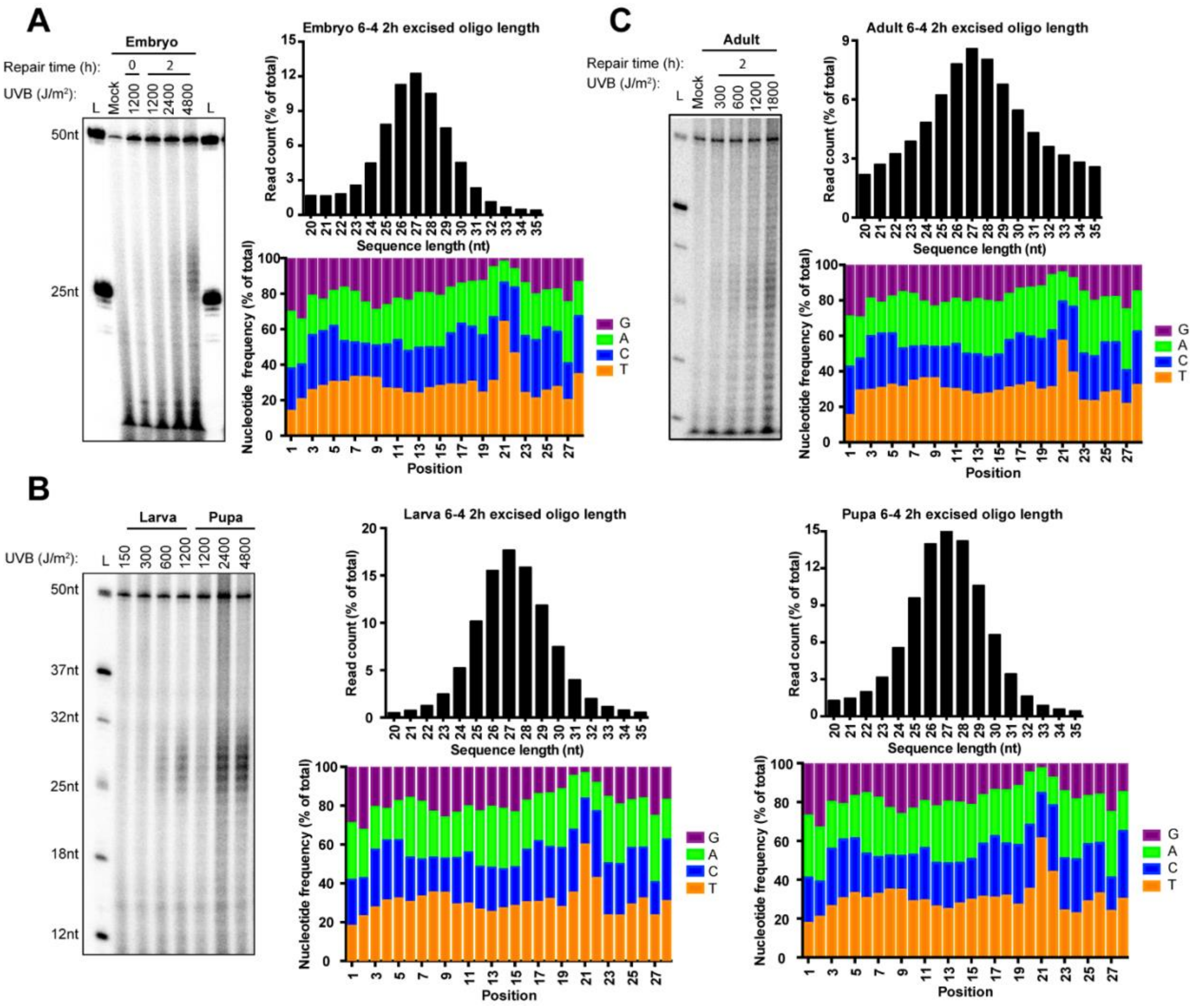

Figure S3. Excision repair of (6-4) PPs in *Drosophila in vivo*. (A) – (C). For each developmental stage, excision assay autoradiograms are shown alongside plots characterizing repair reads generated by XR-seq. **(A)** embryo; **(B)** larva and pupa; **(C)** adult. The comparable dataset for CPD repair is shown in **Fig. 2C**. (6-4) PP repair products are comparable in size to CPD products and both CPD and (6-4) PP repair products are not as readily degraded as repair products in humans and other organisms.

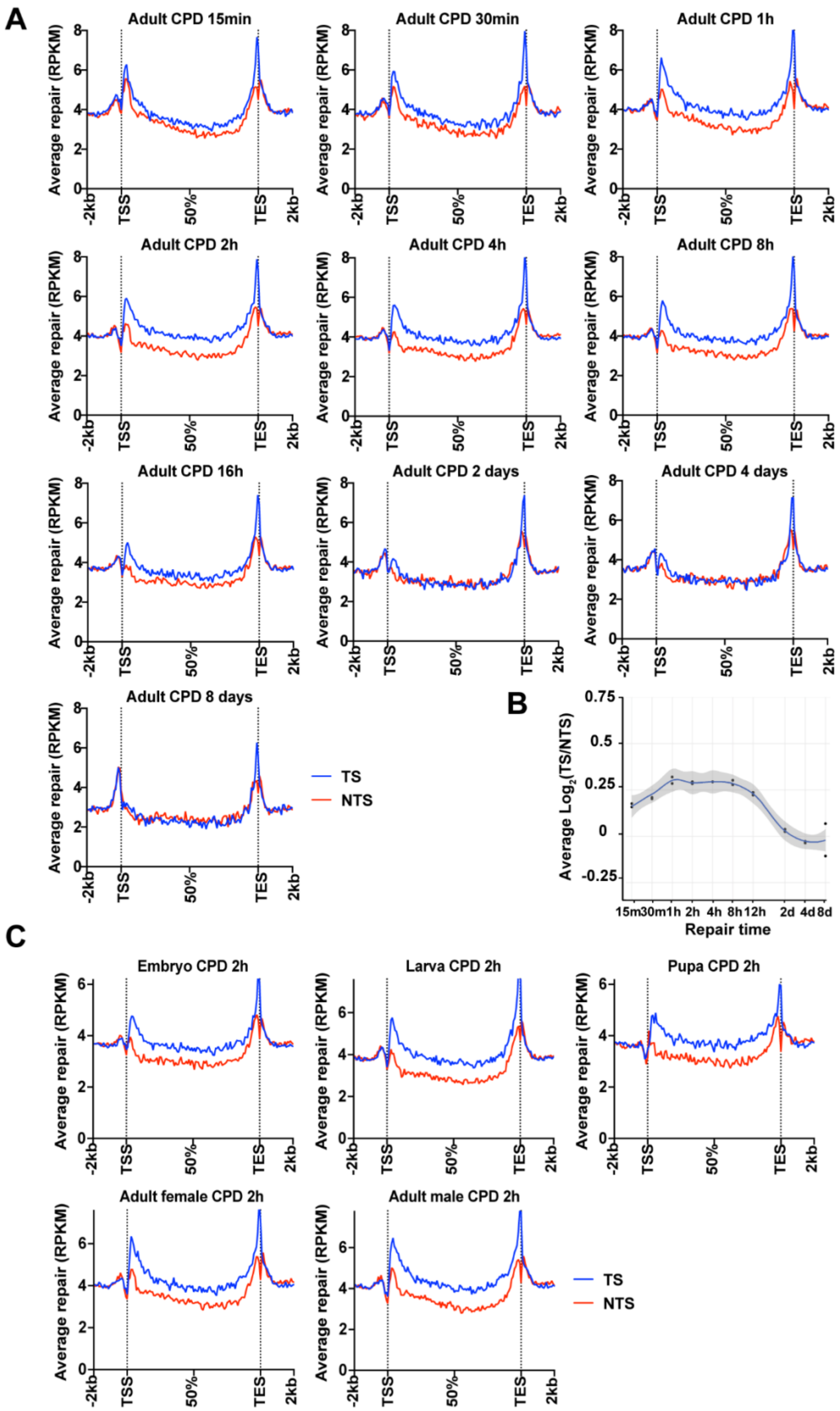
A
Adult CPD 15min
Adult CPD 30min
Adult CPD 1h
Adult CPD 2h
Adult CPD 4h
Adult CPD 8h
Adult CPD 16h
Adult CPD 2 days
Adult CPD 4 days
Adult CPD 8 days
Average repair (RPKM)
-2kb
TSS
50%
TES
2kb
TS
NTS
B
Average Log2(TS/NTS)
0.75
0.5
0.25
0
-0.25
15m30m1h 2h 4h 8h12h 2d 4d 8d
Repair time
C
Embryo CPD 2h
Larva CPD 2h
Pupa CPD 2h
Adult female CPD 2h
Adult male CPD 2h
Average repair (RPKM)
-2kb
TSS
50%
TES
2kb
TS
NTS

Figure S4. Genome-wide analysis of TCR in *Drosophila in vivo*. For each analysis, CPD repair reads were mapped to the genome, and reads from the two strands of each *Drosophila* gene were scaled to a "unit gene" which represents the average repair in each strand of all genes considered. For this analysis, the unit gene was constructed using a *Drosophila* data set of 6218 genes which includes all non-overlapping genes over 1 kb. Thus, these plots include all genes, not only transcribed genes, which are shown in **Fig. 3**. **(A)** Time course for CPD repair in mixed gender adults. **(B)** TCR peaks at approximately 2 h post-UVB and remains high until 8 to 12 h post-UV. **(C)** 2 h CPD repair of embryo, larva, pupa, and adult *Drosophila*. TCR is evident independent of developmental phase.

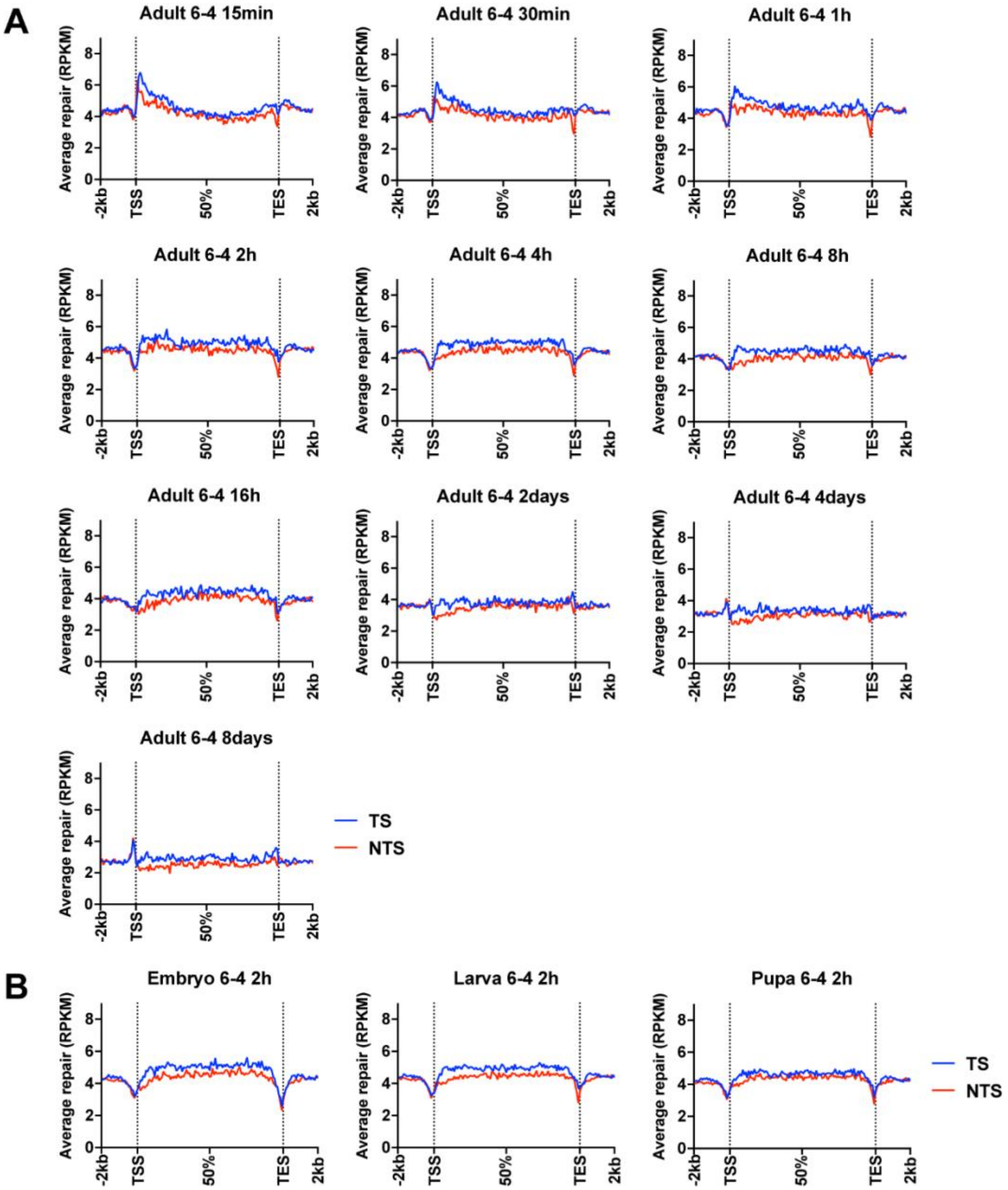

Figure S5. (6-4) PPs are not subject to substantial TCR *in vivo*. (A) Time course for (6-4) PP repair in mixed gender adults. **(B)** 2 h (6-4) PP repair of embryo, larva, pupa and adult in non-overlapping genes over 1 kb in length (6218 genes). (6-4) PP is not substantially repaired by TCR among all developmental phases.

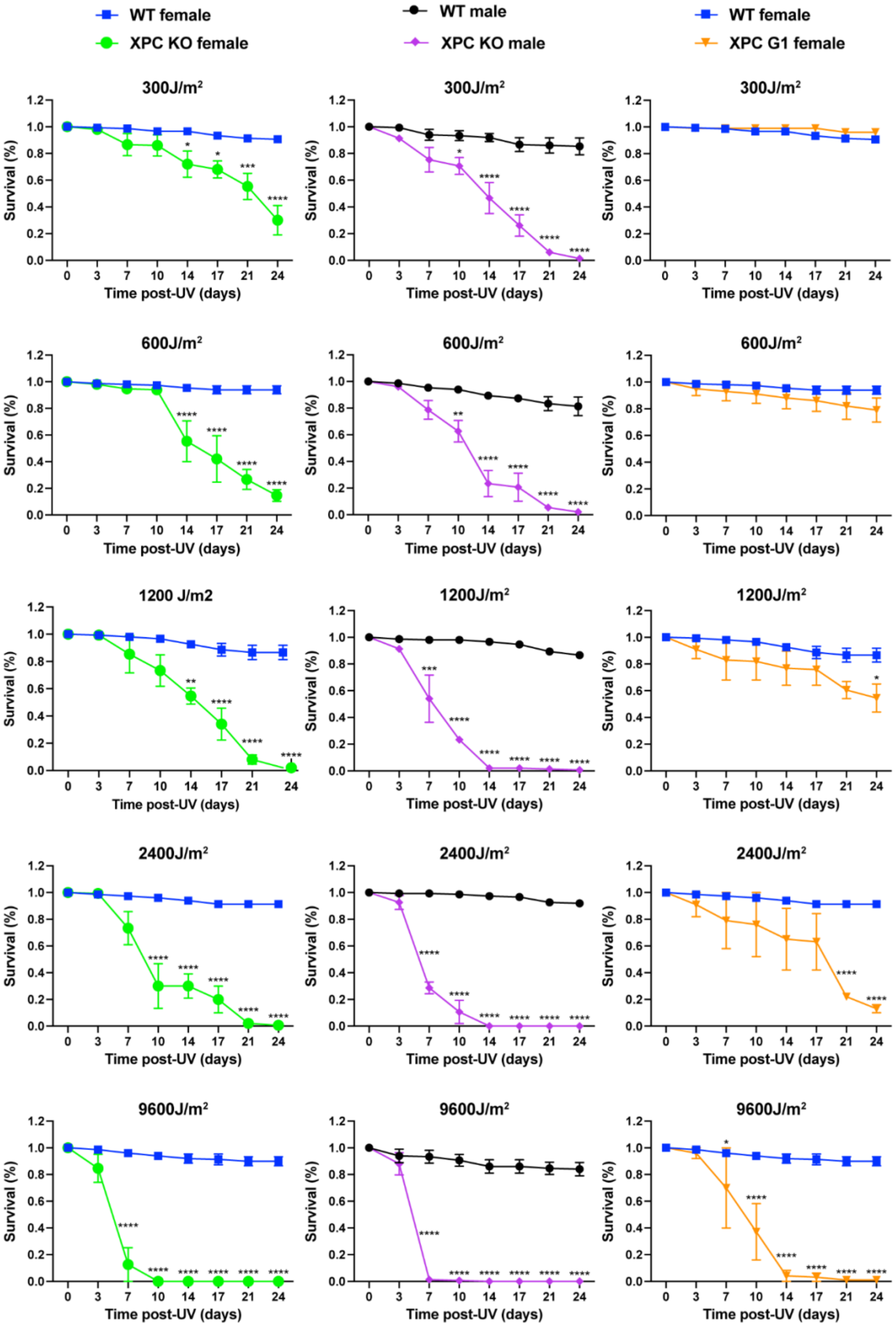
WT female
XPC KO female
WT male
XPC KO male
WT female
XPC G1 female
300J/m²
600J/m²
1200 J/m2
1200J/m²
2400J/m²
9600J/m²
Survival (%)
Time post-UV (days)

Figure S6. **UV sensitivity associated with *XPC* mutations.** Survival of wild-type (W1118), *XPC* mutant (*XPC* G1) and *XPC* knockout (*XPC* KO) adult flies without and with different doses of UVB. *XPC* mutant *(XPC G1)* and *XPC* knockout files are sensitive to UVB damage compared to wild-type flies. Group data were analyzed by 2-way ANOVA (Tukey's multiple comparison test for more than two groups by using GraphPad Prism 8 software) and expressed as means ± SEM, n=3. $^{*}p < 0.05$, $^{**}p < 0.01$, $^{***}p < 0.001$ and $^{****}p < 0.0001$ were considered to be statistically significant.

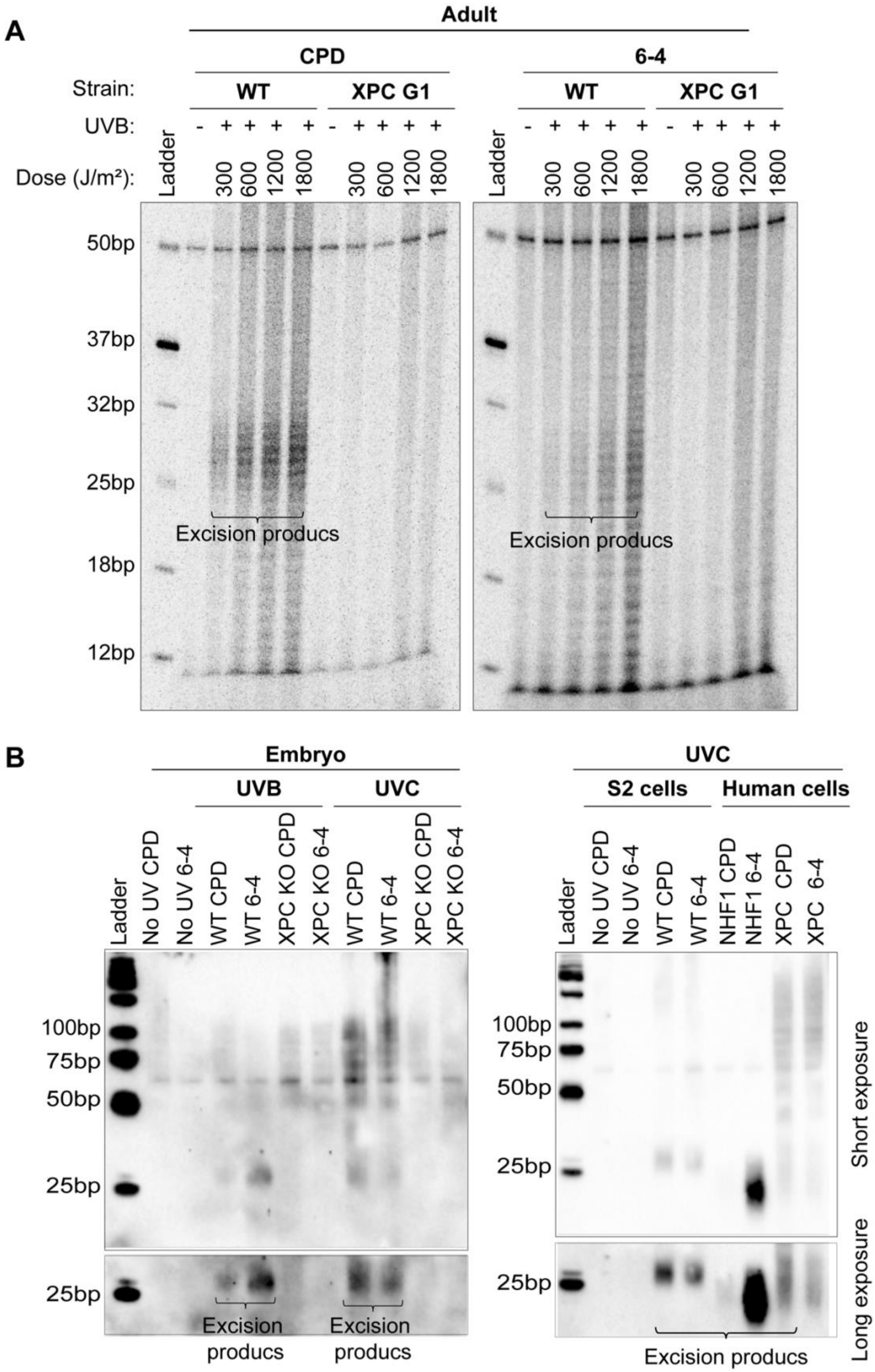
A
Adult
CPD
6-4
Strain:
WT
XPC G1
WT
XPC G1
UVB:
Dose (J/m²):
Ladder
300
600
1200
1800
50bp
37bp
32bp
25bp
18bp
12bp
Excision producs
Excision producs
B
Embryo
UVB
UVC
Ladder
No UV CPD
No UV 6-4
WT CPD
WT 6-4
XPC KO CPD
XPC KO 6-4
100bp
75bp
50bp
25bp
Excision producs
Excision producs
UVC
S2 cells
Human cells
NHF1 CPD
NHF1 6-4
XPC CPD
XPC 6-4
Short exposure
Long exposure
Excision producs

Figure S7. **Absence of excision in the absence of *XPC*.** **(A)** Excision assay of wild-type (W1118) and *XPC* mutant (*XPC* G1) flies 2 h following different doses of UVB. Assays were done with anti-CPD (left panel) and anti-(6-4) PP immunoprecipitation (right panel). **(B)** Excision assay (using biotin labeling) of *Drosophila* wild-type (W1118) and *XPC* knockout (*XPC* KO) embryos 1 h following 2400 J/m^2 of UVB and 500 J/m^2 of UVC (left panel). Excision assay (using biotin labeling) of *Drosophila* S2 cells, wild-type human cells (NHF1) and human *XPC* patient cells (*XPC*) following 20 J/m^2 of UVC (right panel). Assay was done with anti-CPD and anti-(6-4) PP immunoprecipitation as indicated.

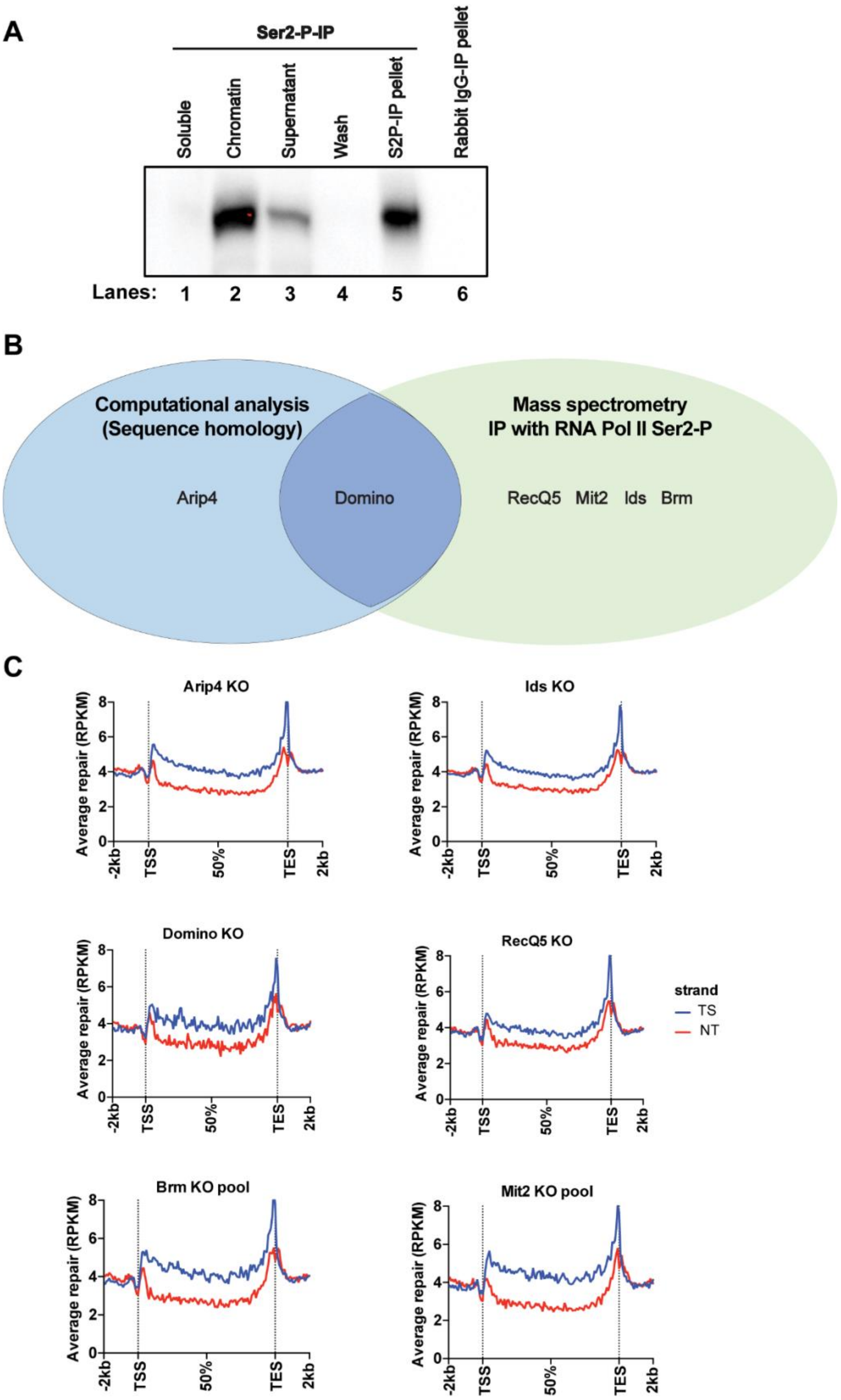
A
Ser2-P-IP
Soluble
Chromatin
Supernatant
Wash
S2P-IP pellet
Rabbit IgG-IP pellet
Lanes: 1 2 3 4 5 6
B
Computational analysis
(Sequence homology)
Mass spectrometry
IP with RNA Pol II Ser2-P
Arip4
Domino
RecQ5 Mit2 Ids Brm
C
Arip4 KO
Ids KO
Domino KO
RecQ5 KO
Brm KO pool
Mit2 KO pool
Average repair (RPKM)
-2kb TSS 50% TES 2kb
strand
TS
NT

Figure S8. Testing potential alternative transcription-repair coupling factors in *Drosophila*. Potential coupling factor candidates were obtained by identifying proteins bound to RNAPII, and by computational methods. **(A)** Isolation of RNAPII with bound proteins. An anti-RNAPII ser2P antibody was used for IP and to detect RNAP on the blot shown. Cells were pelleted to separate Soluble material (lane 1) from the pellet. The pellet was resuspended and lysed, and the resulting Chromatin fraction (lane 2) was separated from soluble material. RNAPII was released from chromatin by treatment with benzonase. The Supernatant fraction following benzonase treatment (Supernatant, lane 3) was incubated with beads containing anti-RNAPII antibodies. Beads were washed (lane 4), and RNAPII was eluted from the beads (lane 5). Nonspecific binding to IgG beads was not detected (lane 6). Proteins bound to eluted RNAPII were identified by mass spectrometry. **(B)** Overlap of putative coupling factors identified by binding to RNAPII and by computational analysis. *Domino, RecQ5, Mit-2, Ids* and *brm* were identified by mass spectrometry as interacting with RNAPII. *RAD54L2* was identified by BLASTing human *CSB* with *Drosophila*. *Dom* was identified by PSI-BLAST of the N-terminus of human *CSB* with *Drosophila*. **(C)** Analysis of data as "unit genes" which reflect the average repair in the TS and NTS strands of *Drosophila* genes was done as in **Fig. 1D**. Mutation of *Arip4*, *Ids*, *Domino*, *RecQ5*, *brm* and *Mit* do not eliminate TCR in S2 cells.

A

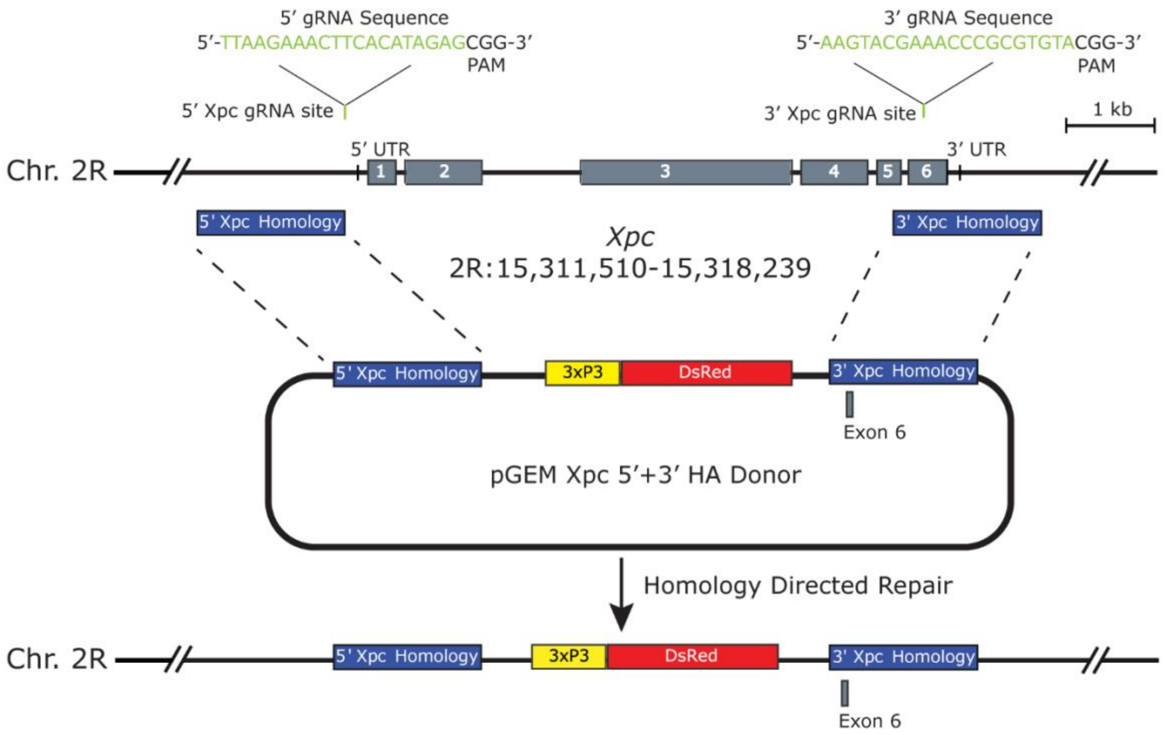

B ΔXPC 5′ Isogenized Male Sequence

ATTCGTTTTGAGAATTCATTTTTGACAATAATGATGCTAACTCGTTTTGTAAATAAACATTTCGCAGATGTGCGTCAGAGCT
GCGTAGTGCTCAAAAATATCGTTTGGAGATAAGTCGATAGCCAAACTAATCGAATATAGAGATGGTGATTTTAGGAGATA
AAAAATGAATGACACTGTTGAAATTATTTAGTCAACACTGTCGGTCAAAAATCTATTAATACGGCTCTATGTGAAGCAATT
GAGCTCATAACTTCGTATAATGTATGCTATACGAAGTTATGTCGACGAATTCGCGGCCGCGAGCTCGCCCGGGGATCTAA
TTCAATTAGAGACTAATTCAATTAGAGCTAATTCAATTAGGATCCAAGCTTATCGATTTCGAACCCTCGACCGCCGGAGTA
TAAATAGAGGCGCTTCGTCTACGGAGCGACAATTCAATTCAAACAAGCAAAGTGAACACGTCGCTAAGCGAAAGCTAAG
CAAATAAACAAGCGCAGCTGAACAAGCTAAACAATCGGGCGGCCGCACTAGAGCCGGTCGCCACCATGAGGTCTTCCAA
GAATGTTATCAAGGAGTTCATGAGGTTTAAGGTTCGCATGGAAGGAACGGTCAATGGGCACGAGTTTGAAATAGAAGGC
GAAGGAGAGGGGAGGCCATACGAAGGCCACAATACCGTAAAGCTTAAGGTAACCAAGGGGGGACCTTTGCCATTTGCT
TGGGATATTTTGTCACCACAATTTCAGTATGGAAGCAAGGTATATGTCAAGCACCCTGCCGACATACCAGACTATAAAAA
GCTGTCATTTCCTGAAGATTTAAATGGGAAAGGGTCATGAACTTTGAAGAC

C ΔXPC 3′ Isogenized Male Sequence

5′-TCGTCTATAATTAGCGCTTTCTATTTTCCCCGATTCGGGCCGCTGCTGCGCTTTTCCGCCTGCTGTTTG
TGGCAAGTGTAGCAGCAGGCTGTGCACGCAGTGTGGCATGCACTTGGCTTTCCACCGTTGGTATCGATTCTCTGGGACGA
TGAGTCATTCCTTTCGGGGCCACAGCATAATCGTTGCCAGCTCACCGAAATGGTGACTTCATTTCTTAACTGCCGTCAAGC
ATGCGATTGTACATACATACATATTTATATATGTACATATTTATGTGACTATGGTAGGTCGATATAATAGCAATCAACGCA
AGCAAATGTGTCAGTCCTGCTTACAGGAACGATTCTATTTAGTAATTTTCGTTGTATAAAGTAATTATGTATGTATGTAAGC
CCCATAAATCTGAAACAATTAGGCAAAACCATGCGAAGCTCTCTGGCGCCTAAAACCCGCGTGTACCGAAACTGGAAGAA
GCTGATCAAGGGTCTCCTCATTCGCGAGCGACTCAAGAAAAAATATAACTTT-3′

Figure S9. **Replacement of endogenous Xpc gene with dsRed via CRISPR/Cas9-induced homology directed repair. (A)** Flies expressing Cas9 in male germline stem cells (under control of the *nanos* promoter) were injected with

plasmids containing guide RNAs 5’ and 3’ to the *Xpc* gene on chromosome 2R (pCFD4 *Xpc* gRNA; under expression of a U6 promoter) and containing donor (template) DNA with 3XP3 (eye) promoter-driven dsRed flanked by homology to regions immediately 5’ and 3’ of Xpc (pGEM *Xpc* 5’ + 3’ HA Donor; injections by Genetivision, Houston, TX). In successful replacement, most of the *Xpc* gene (gray boxes, excluding a small part of exon 6) is excised via Cas9 cutting at the 5’ and 3’ gRNA sites (green) and replaced with dsRed in male germline stem cells by homology directed repair that uses the 5’ and 3’ homologies (blue) contained on the donor plasmid as a template. Males with replacement in their germline stem cells then transmit this modified, Δ*Xpc*:dsRed chromosome to their progeny, and successful replacement is indicated phenotypically by expression of dsRed in eyes. dsRed-positive male progeny were then isogenized and used to make a Δ*Xpc*:dsRed stock. gRNA sequences (green sequence above green boxes) were generated using the flyCRISPR design tool (https://flycrispr.org), with protospacer adjacent motif (PAM) sequences in black. This figure was generated using SnapGene® (with modifications and additions). **(B)** and **(C)** Sanger sequences of PCR products 5’ and 3’ to *Xpc* gene locus, respectively, obtained from the isogenized male used to make the Δ*Xpc*:dsRed stock.

Table S1: CRISPR plasmids and oligo primers used in this study

Primers	Construct	Sequences (5'-3')
Arip4 gRNA fwd	pLib6.4- Arip4 gRNA	GTTCTGTATCCGGATGATCCCAGG
Arip4 gRNA rev	pLib6.4- Arip4 gRNA	AAACCCTGGGATCATCCGGATACA
Lds gRNA fwd	pLib6.4- lds gRNA	GTTCCTTCCTCTTAGTGTTCCTAG
Lds gRNA rev	pLib6.4- lds gRNA	AAACCTAGGAACACTAAGAGGAAG
dom sgRNA fwd	pLib6.4- dom gRNA	GTTCGTATGATGGACTACCCCGCG
dom sgRNA rev	pLib6.4- dom gRNA	AAACCGCGGGGTAGTCCATCATAC
RecQ5 sgRNA fwd	pLib6.4- RecQ5 gRNA	GTTCGCGCATTGTGTTAGCCAATG
RecQ5 sgRNA rev	pLib6.4- RecQ5 gRNA	AAACCATTGGCTAACACAATGCGC
Mi-2 sgRNA fwd	pLib6.4- Mi-2 gRNA	GTTCGCACAGCGTAGCAATGACGA
Mi-2 sgRNA rev	pLib6.4- Mi-2 gRNA	AAACTCGTCATTGCTACGCTGTGC
brm sgRNA fwd	pLib6.4- brm gRNA	GTTCTTTCCACTATCAGTACGACG
brm sgRNA rev	pLib6.4- brm gRNA	AAACCGTCGTACTGATAGTGGAAA

SI References

1. K. Katoh, D. M. Standley, MAFFT multiple sequence alignment software version 7: improvements in performance and usability. *Molecular biology and evolution* **30**, 772-780 (2013).
2. B. Q. Minh *et al.*, IQ-TREE 2: New Models and Efficient Methods for Phylogenetic Inference in the Genomic Era. *Molecular biology and evolution* **37**, 1530-1534 (2020).
3. O. Adebali, D. R. Ortega, I. B. Zhulin, CDvist: a webserver for identification and visualization of conserved domains in protein sequences. *Bioinformatics* **31**, 1475-1477 (2015).
4. S. F. Altschul *et al.*, Gapped BLAST and PSI-BLAST: a new generation of protein database search programs. *Nucleic acids research* **25**, 3389-3402 (1997).
5. Anonymous, UniProt: a worldwide hub of protein knowledge. *Nucleic acids research* **47**, D506-D515 (2019).
